# Atomically dispersed chromium coordinated with hydroxyl clusters enabling efficient hydrogen oxidation on ruthenium

**DOI:** 10.1038/s41467-022-33625-x

**Published:** 2022-10-06

**Authors:** Bingxing Zhang, Baohua Zhang, Guoqiang Zhao, Jianmei Wang, Danqing Liu, Yaping Chen, Lixue Xia, Mingxia Gao, Yongfeng Liu, Wenping Sun, Hongge Pan

**Affiliations:** 1 School of Materials Science and Engineering, State Key Laboratory of Clean Energy Utilization, Zhejiang University, Hangzhou, 310027 PR China; 2 State Key Laboratory of Silicate Materials for Architectures, International School of Materials Science and Engineering, Wuhan University of Technology, Wuhan, 430070 PR China; 3 Institute of Science and Technology for New Energy, Xi’an Technological University, Xi’an, 710021 PR China

**Keywords:** Electrocatalysis, Electrocatalysis, Fuel cells

## Abstract

Overcoming the sluggish kinetics of alkaline hydrogen oxidation reaction (HOR) is challenging but is of critical importance for practical anion exchange membrane fuel cells. Herein, abundant and efficient interfacial active sites are created on ruthenium (Ru) nanoparticles by anchoring atomically isolated chromium coordinated with hydroxyl clusters (Cr_1_(OH)_x_) for accelerated alkaline HOR. This catalyst system delivers 50-fold enhanced HOR activity with excellent durability and CO anti-poisoning ability via switching the active sites from Ru surface to Cr_1_(OH)_x_-Ru interface. Fundamentally different from the conventional mechanism merely focusing on surface metal sites, the isolated Cr_1_(OH)_x_ could provide unique oxygen species for accelerating hydrogen or CO spillover from Ru to Cr_1_(OH)_x_. Furthermore, the original oxygen species from Cr_1_(OH)_x_ are confirmed to participate in hydrogen oxidation and H_2_O formation. The incorporation of such atomically isolated metal hydroxide clusters in heterostructured catalysts opens up new opportunities for rationally designing advanced electrocatalysts for HOR and other complex electrochemical reactions. This work also highlights the importance of size effect of co-catalysts, which should also be paid substantial attention to in the catalysis field.

## Abstract (web summary)

Overcoming the sluggish kinetics of hydrogen oxidation reaction in alkaline media is of critical importance for anion exchange membrane fuel cells. Here the authors report atomically dispersed chromium coordinated with hydroxyl clusters on ruthenium nanoparticles for accelerated hydrogen oxidation reaction.

## Introduction

Anion exchange membrane fuel cells (AEMFCs) possess the promising application foreground over proton-exchange membrane fuel cells (PEMFCs) as nonprecious metal electrocatalysts can be substituted for the platinum-group-metal (PGM)-based electrocatalysts in the cathodic oxygen reduction reaction^1–3^. However, the anodic hydrogen oxidation reaction (HOR) of AEMFCs still relies on PGM-based catalysts for achieving desirable performance. The much more sluggish HOR kinetics in alkaline media further makes AEMFCs require higher loading of PGM-based catalysts to achieve the ideal power density compared to PEMFCs^4–7^. In this respect, ever-increasing effort has recently been devoted to developing active alkaline HOR electrocatalysts, particularly non-PGM-based catalysts, and to uncovering the intrinsic reasons for the inferior HOR activity in alkaline media^8–13^. Despite considerable progresses have been achieved, breakthrough in terms of developing robust alkaline HOR electrocatalysts and in-depth understanding of the alkaline HOR mechanism is still urgent at this stage.

Electrocatalysts with an optimized surface/interface or electronic structure will largely balance the adsorption of intermediates (e.g., H, OH, and CO), thus enhancing their hydrogen electrocatalysis and anti-poisoning performance^14–16^. In this regard, various approaches such as doping or alloying^17–24^ and heterostructure engineering^25–31^ have been widely proposed to enhance the activity and durability of HOR electrocatalysts. However, most of these strategies can only partially resolve the problems of alkaline HOR. For example, the nanoscale heterostructure engineering focuses on the bifunctional mechanism^25^, which merely optimizes the OH binding energy (OHBE) with the help of the transition metal (hydr)oxides but fails to tune the H binding energy (HBE), and hence provides very limited interfacial active sites. Although the alloying method can regulate the HBE by altering the electronic structure and provide abundant active sites^17,23^, the doped metallic atom possesses less oxophilicity for OH^-^ adsorption as compared with its oxide counterpart^23^. Therefore, it is highly important to develop an efficient strategy to simultaneously optimizing HBE and OHBE with groundbreaking insights for innovating HOR pathway.

**Fig. 1:**
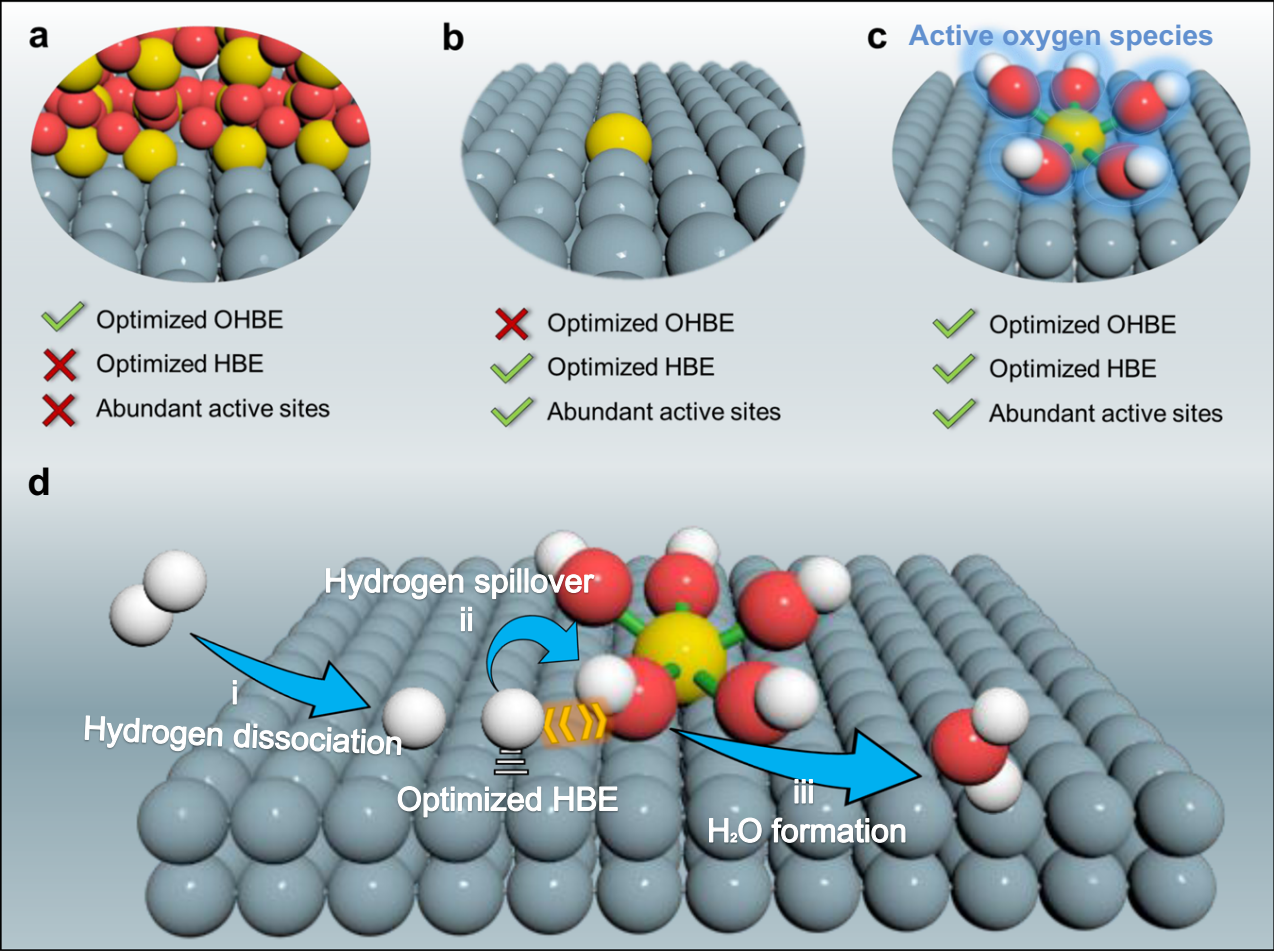
Scheme illustration of the proposed catalyst design strategy for HOR. Typically reported heterostructure (**a**) and single-atom alloy (**b**) catalysts for HOR. **c** The structure of Ru–Cr_1_(OH)_x_ catalyst. **d** Proposed HOR mechanism for Ru–Cr_1_(OH)_x_ catalyst. Color: Gray, Ru; yellow, Cr; red, O; white, H.

In this work, combining the structural features of single-atom alloy and heterostructure catalysts, we design a distinctive Ru-based catalyst anchored by atomically dispersed chromium coordinated with hydroxyl clusters (Ru–Cr_1_(OH)_x_) for accelerated HOR kinetics (Fig. 1). Particularly, the isolated Cr_1_(OH)_x_ clusters, as the co-catalyst, can maximize interfacial active sites and create abundant active oxygen species for optimizing HBE and promoting hydrogen or CO spillover from Ru to Cr_1_(OH)_x_. Based on the isotope-labeling experiments, the original oxygen species from Cr_1_(OH)_x_ are also confirmed to react with adsorbed hydrogen for a faster hydrogen oxidation kinetics. As results, the HOR activity of Ru–Cr_1_(OH)_x_ is 50-fold higher than the unmodified Ru. In addition, the isolated Cr_1_(OH)_x_ clusters are also conducive to removing the CO molecules and supressing the aggregation of Ru species during HOR, eventully delivering excellent CO-tolerance and long-durability performance. The proposed strategy is superior to typical designs of (hydr)oxide nano-modifiers and alloy catalyst, also presents fundamentally different HOR mechanism compared with conventional insights centered on surface metal sites.

## Results and discussion

### Material synthesis and characterization

**Fig. 2:**
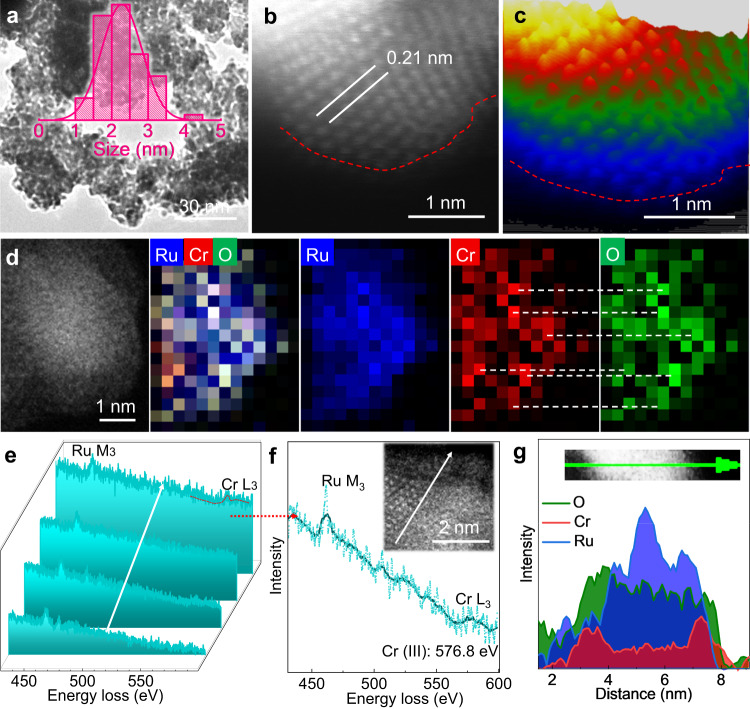
Structural characterizations of Ru–Cr_1_(OH)_x_. **a** TEM image. Inset is the size distribution of nanoparticles. High-resolution HAADF-STEM image (**b**) and the corresponding pseudo-color surface plot (**c**). **d** EELS mapping images. The line scanning EELS spectra (**e**) and the enlarged EELS spectra for the edge of Ru particle (**f**). Inset in **f** is the line scanning path in the STEM-HAADF image. **g** EDS line-scanning profile across the nanoparticle and the corresponding HAADF-STEM image (inset).

The Ru–Cr_1_(OH)_x_ catalyst was synthesized by a sequential reduction and deposition method followed by freeze drying (See Methods), during which the metallic Ru nanoparticles were rapidly formed and concomitantly anchored by the Cr_1_(OH)_x_ clusters (Supplementary Fig. 1). High-angle annular dark-field scanning transmission electron microscopy (HAADF-STEM), scanning electron microscopy (SEM) and transmission electron microscopy (TEM) images (Fig. 2a, Supplementary Figs. 2 and 3) of the Ru–Cr_1_(OH)_x_ catalyst show an interconnected network structure with an aerogel morphology and average particle size of 2.4 nm. The X-ray diffraction (XRD) pattern (Supplementary Fig. 4) shows that Ru–Cr_1_(OH)_x_ presents the sole metallic Ru structure and no Cr-based crystalline phase can be detected. High-resolution HAADF-STEM images (Fig. 2b, c) show the presence of a less electron-dense layer on the surface of metallic Ru nanoparticles, which is composed of the atomically dispersed Cr species with low weight loading on Ru surface. The interplanar distances of 0.21 nm is assigned to the (002) planes of Ru (Fig. 1b). Energy-dispersive X-ray spectrometry (EDS, EDS, Supplementary Fig. 5) elemental mapping and electron energy loss spectroscopy (EELS, Fig. 1d) mapping images show the Cr, Ru and O species are homogenously dispersed in both entire catalysts and one single nanoparticle. It can also be observed that the distributions of Cr and O species are very consistent and more obvious than that of Ru signal on the edge of nanoparticle (Fig. 2d). Furthermore, the signals of Cr and O species are not beyond the scope of Ru nanoparticle, suggesting it stays as the oxygen coordinated Cr_1_ species on Ru surface but not a multiple-atom overlapped shell, which is further confirmed by the isolated Cr signal in the selected local EELS mapping image (Supplementary Fig. 6). For the chemical state of Cr, the obvious Cr (III) signal in EELS collected on the edge of Ru particle (Fig. 2e, f) confirms it is trivalent Cr_1_ species anchored on Ru surface. The EDS line-scanning profile (Fig. 2g) also clearly evidences the Ru surface is covered with Cr and O species. Inductively coupled plasma-optical emission spectroscopy (ICP-OES, Supplementary Table 1) demonstrates the amount of Cr in Ru–Cr_1_(OH)_x_ is 1.1 wt% (denoted as Ru–Cr_1_(OH)_x_−1.1). Time-of-fight secondary ion mass spectrometry (ToF-SIMS) spectra were further performed and reveal that single Cr (III) ion peaks are observed while other Cr–O multimers cannot be obviously detected, again confirming the atomically dispersed Cr (III) species on Ru nanoparticles^32^ (Supplementary Fig. 7). The above results together confirm the atomically dispersed Cr (III) coordinated with hydroxyl/oxygen clusters is successfully constructed on Ru nanoparticle for Ru–Cr_1_(OH)_x_, which will be further dissected by other technologies below. In addition, pure Ru nanoparticles (NPs) and other Ru–Cr_1_(OH)_x_−*y* samples (y% is the weight fraction of Cr) of Ru–Cr_1_(OH)_x_−0.6 and Ru–Cr_1_(OH)_x_−2.2 were also synthesized (Supplementary Figs. 8–11).

**Fig. 3:**
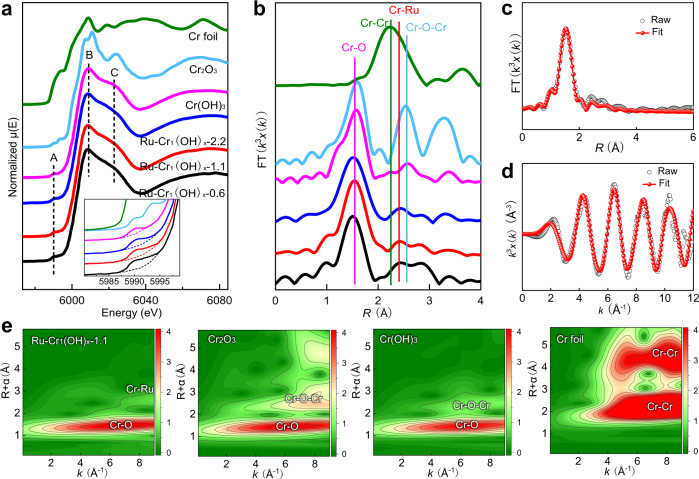
XAFS measurements of different samples at the Cr K-edge. **a** XANES spectra. The inset displays a magnified view of the pre-edge peak A. **b** *k*^3^-weighted FT-EXAFS spectra in *R*-space. EXAFS fitting curves of Ru–Cr_1_(OH)_x_−1.1 in *R* (**c**) and *k* (**d**) space. **e** WT for the k^3^-weighted EXAFS of different samples.

X-ray absorption spectroscopy was further performed to uncover the detailed structure of the atomically dispersed Cr_1_(OH)_x_ species. As shown in Fig. 3a, the Cr K-edge X-ray absorption near-edge structure (XANES) spectra indicate that the three as-synthesized catalysts show a stronger pre-edge peak A (inset in Fig. 3a) and a larger intensity ratios for peaks B and C (*I*_B_/*I*_C_) than those for Cr(OH)_3_. The profile differences suggest the existence of more distorted coordination structure of Cr–OH in Ru–Cr_1_(OH)_x_ due to the atomic dispersion characteristic^33^. In the Fourier transforms of the extended X-ray absorption fine-structure (FT-EXAFS) spectra, the peak at 2.42 Å between Cr-Cr (2.25 Å) and Cr–O–Cr (2.54 Å) can be well assigned to the Cr-Ru coordination (Fig. 3b). This is further confirmed by the least-squares EXAFS curve-fitting analysis for Cr–O, Cr–Ru and Cr–Cr coordination (Fig. 3c,d), which respectively shows the coordination numbers of 4.5, 1.5 and 0.6 for Ru–Cr_1_(OH)_x_−1.1 with appropriate bond length and fitting parameters (Supplementary Table 2). The FT-EXAFS spectra along with the fitting results reveal that most Cr species are atomically dispersed on the Ru nanoparticles, whereas multimer chromium-oxygen (Cr–O–Cr) is less populated (<10%, Supplementary Table 2)^34^. As expected, the ratio of the isolated Cr_1_(OH)_x_ is decreased with increasing the loading of Cr species in Ru–Cr_1_(OH)_x_ samples (Supplementary Fig. 12 and Supplementary Table 2). Wavelet transform (WT) of EXAFS (Fig. 3e, Supplementary Fig. 13) can help distinguish the type of backscattering atoms. Ru–Cr_1_(OH)_x_−1.1 exhibits one weak signal at >8.0 Å^−1^ in *k* space that corresponds to the Cr-Ru coordination because of the higher atomic number of Ru. In contrast, the Cr–Cr in Cr foil and Cr–O–Cr in bulk Cr_2_O_3_ and Cr(OH)_3_ at 7–8 Å^−1^ in *k* space are not observed in Ru–Cr_1_(OH)_x_−1.1, further suggesting that the Cr_1_(OH)_x_ species are atomically anchored on the metallic Ru surface, consistent with the above quantitative EXAFS analysis. On the other hand, the Ru–K-edge XANES and FT-EXAFS spectra of Ru–Cr_1_(OH)_x_ samples show comparable metallic characteristic with that of pure Ru nanoparticles (Supplementary Fig. 14). In addition, the Cr K-edge XANES simulation for Ru–Cr_1_(OH)_x_−1.1 was also carried out, and the calculated XANES matches very well with the collected spectroscopy (Supplementary Fig. 15), further verifying the proposed structure of atomically dispersed Cr_1_(OH)_x_ species on Ru surface.

**Fig. 4:**
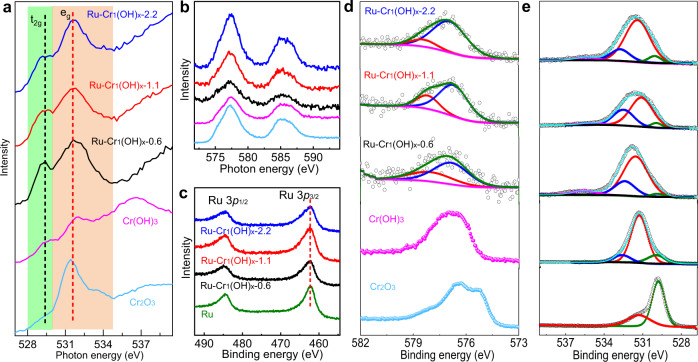
Fine structural characterizations. O K (**a**) and Cr L_II, III_ (**b**) edges NEXAFS spectra. Ru 3*p* (**c**), Cr 2*p* (**d**) and O 1*s* (**e**) XPS spectra.

As shown in the near-edge X-ray absorption fine-structure (NEXAFS) spectra collected at O K edge (Fig. 4a), the characteristic peaks between 528-535 eV can be attributed to the spectroscopic excitations to the hybridized state of O 2*p* and Cr-3*d*, which can be divided into the *t*_2g_ and *e*_g_ orbital peaks^35^. The pronounced feature at 529.2 eV is ascribed to the Cr-deficient sites and disordered environment due to the oxygen-rich Cr_1_(OH)_x_ clusters in Ru–Cr_1_(OH)_x_^36^. In addition, both the intensity and position of the e_g_ orbital peaks for Ru–Cr_1_(OH)_x_ samples exhibit different profiles compared to Cr_2_O_3_ and Cr(OH)_3_, confirming the distinct oxygen species in single Cr_1_(OH)_x_ clusters. The Cr L_II,III_-edge NEXAFS spectra show the anchored Cr species present an identical Cr (III) state as compared to Cr_2_O_3_ and Cr(OH)_3_ (Fig. 4b). X-ray photoelectron spectra (XPS) show that the ratios of Cr to Ru are larger than those determined by ICP, because Cr_1_(OH)_x_ are anchored on Ru surface (Supplementary Table. 1). The Ru 3*p*_3/2_ binding energy for all Ru-based catalysts is around 462.3 eV, indicating that the oxidation state of Ru is not obviously changed after the incorporation of Cr species (Fig. 4c), in good agreement with the XAFS results. As shown in Fig. 4d, the Cr 2*p*_3/2_ binding energy in the Ru–Cr_1_(OH)_x_ samples shifts positively by ∼0.9 eV and ~0.4 eV compared with those of Cr_2_O_3_ and Cr(OH)_3_, respectively. The Cr 2*p*_3/2_ XPS spectra of Ru–Cr_1_(OH)_x_ samples can be fitted into two chemical states. The one located at 576.8 eV is assigned to Cr (III) trivalent hydroxide state and the other one at 578.3 eV is considered to be Cr (III) coordinated with additional hydroxyl ligands in Cr_1_(OH)_x_^37^. More hydroxyl ligands are attached to Cr in Cr_1_(OH)_x_, which can well elucidate the increased binding energy of Cr 2*p*_3/2_. The fraction of the peak at 578.3 eV is increased from Ru–Cr_1_(OH)_x_−2.2 to Ru–Cr_1_(OH)_x_−0.6, consistent with the result that the atomically dispersed Cr species are coordinated with more hydroxyl ligands. Accordingly, Ru–Cr_1_(OH)_x_ samples exhibit a broad O 1 *s* peak at ~532.0 eV, which exhibits a positive shift compared to the other two references (Fig. 4e) and can be further divided into Cr–O–Cr (~529.9 eV), Cr-OH (~531.2 eV) and adsorbed H_2_O (~532.6 eV). The content of Cr–O–Cr decreased dramatically from Cr_2_O_3_ (70.0%) and Cr(OH)_3_ (14.4%) to Ru–Cr_1_(OH)_x_−0.6 (3.0%), Ru–Cr_1_(OH)_x_−1.1 (4.7%) and Ru–Cr_1_(OH)_x_−2.2 (6.3%), further validating the Cr–O–Cr bonds are largely broken so that more exposed oxygen atoms are formed in Cr_1_(OH)_x_ clusters. All of the above results confirm the presence of abundant active oxygen species in the atomically dispersed Cr_1_(OH)_x_ clusters, which would enable a unique catalytic process with faster kinetics compared with the typical nanoscale metal-support interfaces.

### Electrocatalytic performance toward alkaline HOR

**Fig. 5:**
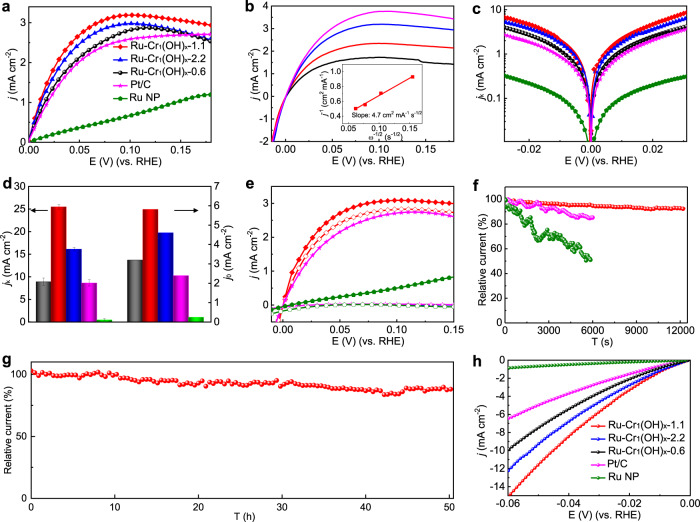
Electrochemical HOR performance. **a** HOR polarization curves of different catalysts. **b** Polarization curves of Ru–Cr_1_(OH)_x_−1.1 at different rotation speeds (*ω*) of 400, 900, 1600, and 2500 rpm (from bottom to top). Inset is the Koutecky–Levich plot at an overpotential of 25 mV. **c** Calculated HOR kinetic current densities (*j*_k_) vs. potential plots recorded from **a**. **d** Comparison of *j*_k_ and *j*_0_ of Ru–Cr_1_(OH)_x_−0.6 (black), Ru–Cr_1_(OH)_x_−1.1 (red), Ru–Cr_1_(OH)_x_−2.2 (blue), Ru NPs (green), and Pt/C (pink). Error bars show as mean s.d. **e** HOR polarization curves for Ru–Cr_1_(OH)_x_−1.1 (red), Ru NPs (green) and Pt/C (pink) in H_2_-saturated 0.1 M KOH without (solid lines) and with (dashed lines) the presence of 20,000 p.p.m. CO. **f** Relative current-time chronoamperometry of different catalysts at an overpotential of 50 mV operated on RDE. **g** Relative current-time chronoamperometry response of Ru–Cr_1_(OH)_x_−1.1 catalyst loaded on carbon fiber paper at an overpotential of 50 mV in a H-type cell. **h** HER polarization curves in 0.1 M KOH solution at a rotating speed of 1600 rpm.

The electrocatalytic performance was evaluated by a rotating disk electrode (RDE) with a standard three-electrode system. The HOR polarization curves were obtained in a H_2_-saturated 0.1 M KOH electrolyte at 25 °C and ambient pressure with a rotating speed of 1600 rpm. The current densities of Ru–Cr_1_(OH)_x_ catalysts are significantly enhanced compared with pure Ru nanoparticles (Fig. 5a), indicating the atomically dispered Cr_1_(OH)_x_ species play a vital role in improving the HOR activity. In particular, the Ru–Cr_1_(OH)_x_−1.1 catalyst has much lower half-wave potential (20 mV) than those of Pt/C (27 mV) and Ru NPs (88 mV) (Supplementary Table 3). With increasing the rotation speed (*ω*), the current density of Ru–Cr_1_(OH)_x_−1.1 presents a linear relationship (Levich plot) between the *j*^−1^ and *ω*^−1/2^ with calculated slope of 4.7 cm^−2^ mA^−1^·s^−1/2^ (Fig. 5b), which is close to the theoretical value of 4.87 cm^2^ mA^−1^ s^−1/2^ for the two-electron HOR. HOR/HER Tafel plots of the kinetic current density (*j*_k_) are then calculated based on the Koutecky–Levich equation (Fig. 5c), indicating the Ru–Cr_1_(OH)_x_−1.1 shows the highest kinetic activity over the potential range among all the presented catalysts. Figure 5d shows the *j*_k_ at 50 mV and the exchange current densities (*j*_0_) extracted by fitting *j*_k_ according to the Butler-Volmer equation. The Ru–Cr_1_(OH)_x_−1.1 catalyst possesses the highest kinetic current density of 25.5 mA cm^−2^, which is 51, 3.0, 2.9 and 1.6 times as high as those of Ru NPs (0.5 mA cm^−2^), Pt/C (8.6 mA cm^−2^), Ru–Cr_1_(OH)_x_−0.6 (8.9 mA cm^−2^) and Ru–Cr_1_(OH)_x_−2.2 (16.1 mA cm^−2^), respectively. The geometric *j*_0_ of Ru–Cr_1_(OH)_x_−1.1 (5.8 mA cm^−2^) is also significantly higher than those of Ru NPs (0.26 mA cm^−2^), Pt/C (2.4 mA cm^−2^), Ru–Cr_1_(OH)_x_−0.6 (3.2 mA cm^_2^) and Ru–Cr_1_(OH)_x_−2.2 (4.6 mA cm^−2^). The intrinsic HOR activities were further normalized by the electrochemically active surface area (ECSA) that was determined by copper underpotential deposition (Cu-UPD) cyclic voltammetry (Supplementary Figs. 16 and 17 and Supplementary Table 3), and the Ru–Cr_1_(OH)_x_−1.1 catalyst still exhibits the highest specific activity. The above results strongly suggest that incorporation of appropriate Cr_1_(OH)_x_ clusters on Ru surface can sigificantly contribute to the improvement of HOR activity, while the formation of multimer chromium-oxygen clusters in Ru–Cr_1_(OH)_x_−2.2 is unfavorable to activity enhancement.

CO anti-poisoning ability is another important concern for HOR electrocatalysts in practical AEMFCs fueled by hydrogen from fossil fuels. Interestingly, Ru–Cr_1_(OH)_x_−1.1 shows exceptional HOR activity in the presence of CO, whereas both Pt/C and Ru NPs lose the HOR activity rapidly under the same test condition (Fig. 5e and Supplementary Fig. 18). The results indicate that the atomically dispered Cr_1_(OH)_x_ species play an important role in preventing CO poisoning in the HOR process. The large amount of active oxygen species in Cr_1_(OH)_x_ clusters could assist the fast oxidation of CO on Ru surface, resulting in notable CO-tolerance performance. With regard to the catalytic durability, the HOR current density for Ru–Cr_1_(OH)_x_−1.1 at 50 mV shows a slight decay of 7% during continuous RDE test for 12000s (Fig. 5f), and, notably, the morphology, composition and structure of the catalyst were well maintained after durablity evaluation (Supplementary Figs. 19 and 20). By contrast, Ru NPs and Pt/C suffer obvious activity degradation of 45% and 15% in current density after only 6000 s, resepctively, mainly resulting from the agglomeration of active sites during the test (Supplementary Fig. 21). The Ru–Cr_1_(OH)_x_−1.1 catalyst was further deposited onto carbon papers in order to mitigate the loss of catalyst under the RDE testing condition^10^, and only shows ~12% activity loss after 50-h durability test in a H-type cell (Fig. 5g). The results imply that the presence of Cr_1_(OH)_x_ clusters can efficiently suppress the agglomeration and degradation of active sites during the HOR process^31^. As shown in Fig. 5h, the Ru–Cr_1_(OH)_x_−1.1 also exhibits the highest HER catalytic activity with an overpotential of 45 mV to reach 10 mA cm^−2^, which is superior to those of Ru–Cr_1_(OH)_x_−2.2 (53 mV) and Ru–Cr_1_(OH)_x_−0.6 (60 mV).

### Mechanism investigation

**Fig. 6:**
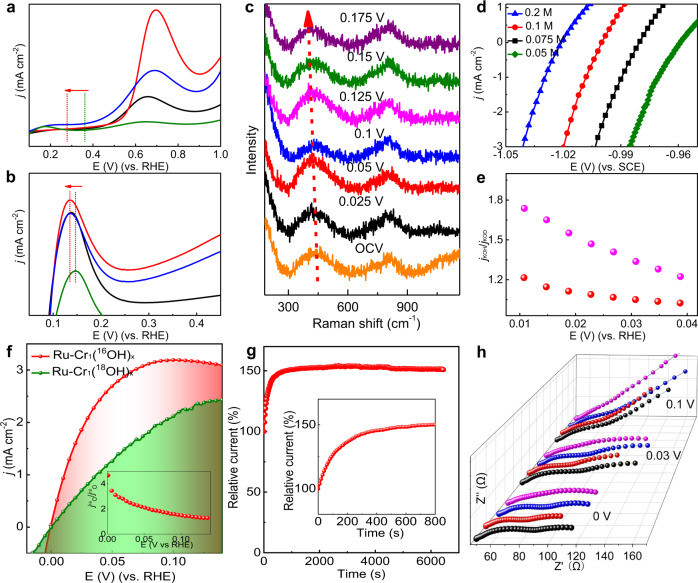
Mechanism investigation. CO-stripping (**a**) and H_upd_ (**b**) measurements for Ru–Cr_1_(OH)_x_−0.6 (black), Ru–Cr_1_(OH)_x_−1.1 (red), Ru–Cr_1_(OH)_x_−2.2 (blue), and Ru NPs (green). **c** In situ Raman spectra of Ru–Cr_1_(OH)_x_−1.1 at different overpotentials. **d** LSV curves of Ru–Cr_1_(OH)_x_−1.1 for HOR in different pH conditions. **e** H/D isotope effect analysis (*j*_KOH_/*j*_KOD_ vs. potential) for Ru–Cr_1_(OH)_x_−1.1 (red) and Pt/C (pink) in 0.1 M KOH/KOD electrolyte. **f** HOR polarization curves of Ru–Cr_1_(^18^OH)_x_ and Ru–Cr_1_(^16^OH)_x_ catalysts, inset is the corresponding current ratios of Cr_1_(^16^OH)_x_ and Ru–Cr_1_(^18^OH)_x_. **g** Relative current-time chronoamperometry of Ru–Cr_1_(^18^OH)_x_ catalysts at an overpotential of 50 mV. **h** Operando EIS of Ru–Cr_1_(OH)_x_−0.6 (black), Ru–Cr_1_(OH)_x_−1.1 (red), Ru–Cr_1_(OH)_x_−2.2 (blue), and Pt/C (pink).

To reveal the mechanism of the highly catalytic activity of Ru derived from the Cr_1_(OH)_x_ clusters, a series of experiments including electrochemistry, isotope-labeling and in situ Raman spectroscopy were conducted. CO stripping experiment (Fig. 6a and Supplementary Fig. 22) displays that the onset potential of CO stripping for Ru–Cr_1_(OH)_x_ catalysts (~0.25 V) is substantially lower than those of Ru NPs (0.36 V) and Pt/C (0.54 V), suggesting the enhanced OHBE of Ru–Cr_1_(OH)_x_ samples and the largely accelerated CO oxidation triggered by the hydroxyl on Cr_1_(OH)_x_^38^. On the other hand, the position of hydrogen underpotential deposition (H_upd_) peak (Fig. 6b) shows that the desorption peak position (~0.135 V) for Ru–Cr_1_(OH)_x_ catalysts is lower than those of Ru NPs (0.147 V) and Pt/C (0.291 V), revealing the weakened Ru-H binding^39^. The above HBE influenced by the Cr_1_(OH)_x_ clusters was further explored by in situ Raman spectroscopy (Fig. 6c, Supplementary Fig. 23). The broad feature at 810 cm^−1^ can be ascribed to symmetric and anti-symmetric Cr-OH stretching, while the feature at 440 cm^−1^ is assigned to Cr–O–H bending mode^40^. With increasing the overpotential from open circuit voltage (OCV) to 175 mV during the HOR process, a red shift becomes more and more pronounced for the peak associated with Cr–O–H bending mode, which could be attributed to the hydrogen spillover from Ru surface to the active oxygen species of Cr_1_(OH)_x_.^41,42^ The active oxygen species of Cr_1_(OH)_x_ clusters for shuffling the hydrogen is further confirmed by the increased pH of ultrapure water after dispersing Ru–Cr_1_(OH)_x_ powders (Supplementary Fig. 24)^36^. The above results indicate the unique oxygen species in Cr_1_(OH)_x_ can facilely interact with the hydrogen adsorbed on Ru surface, resulting in optimized HBE, which simultaneously promotes the Volmer step.

The role of oxygen species in HOR process was further investigated in detail. The Ru–Cr_1_(OH)_x_−1.1 catalyst exhibits a similar kinetic activity of HOR in 0.05 M to 0.2 M KOH (Fig. 6d), which is different from the behaviors of Pt/C (Supplementary Fig. 25), suggesting the Cr_1_(OH)_x_ clusters have strong capacity in providing enough reactive hydroxyl species in alkaline media. The HOR activity of Ru–Cr_1_(OH)_x_ catalyst has an H/D isotope effect of 1.0–1.2 influenced by the applied potentials (Fig. 6e), whereas Pt/C possesses an H/D isotope effect of 1.2–1.7. The results indicate that the OH^-^ substituted by OD^-^ in the electrolyte will reduce the HOR activity because hydroxyl participates in the HOR process^43^. However, the Ru–Cr_1_(OH)_x_ catalyst is less affected in the process, implying the Cr_1_(OH)_x_ clusters can also adsorb OD^-^ efficiently for further reaction due to their unique role in constructing active oxygen species, which is consistent with the results of pH dependence of HOR activity. The active oxygen species in Cr_1_(OH)_x_ clusters for HOR were further examined via kinetic isotope effect (KIE) studies by ^18^O-labeled Cr_1_(^18^OH)_x_ in the catalysts, which were obtained by substituting H_2_^18^O for H_2_O in the synthesis process. The HOR kinetics of Ru–Cr_1_(^18^OH)_x_ is much slower than that of Ru–Cr_1_(^16^OH)_x_ (Fig. 6f), indicating that the hydroxyl species of Cr_1_(^18^OH)_x_ participate in the rate-determining step of the HOR since their mass increases upon the substitution. When the ^18^OH in the clusters is gradually substituted by the ^16^OH during the HOR process, the HOR kinetics would be recovered accompanied with the formation of H_2_^18^O (Fig. 6g, Supplementary Fig. 26), further confirming the original active hydroxyl in the Cr_1_(OH)_x_ clusters participates in the reaction step of HOR.

The higher HOR activity of Ru–Cr_1_(OH)_x_−1.1 compared with the other two samples (Ru–Cr_1_(OH)_x_−0.6 with lower density of Cr_1_(OH)_x_ and Ru–Cr_1_(OH)_x_−2.2 with multimer chromium-oxygen clusters as confirmed in Fig. 3) is investigated by operando electrochemical impedance spectroscopy (EIS). Ru–Cr_1_(OH)_x_−1.1 shows a smaller low-frequency-dominant specific resistance from low to high overpotentials (Fig. 6h), suggesting the higher activity of per active site. The Nyquist plots are fitted by a double-parallel equivalent circuit model (Supplementary Figs. 27 and 28 and Supplementary Table 4)^44^. The first and second parallel component represent the charge transfer resistance R_1_ and hydrogen adsorption resistance R_2_. EIS-derived Tafel curves of charge transfer kinetics and hydrogen adsorption kinetics clearly show that the R_1_ and R_2_ of Ru–Cr_1_(OH)_x_−1.1 are lower than the other two samples (Supplementary Fig. 29). It also suggests that the Tafel slopes of logR_1_ and logR_2_ vs. overpotential for Ru–Cr_1_(OH)_x_−1.1 are lower and close to those of Ru–Cr_1_(OH)_x_−0.6 and Ru–Cr_1_(OH)_x_−2.2, respectively, indicating the Ru–Cr_1_(OH)_x_−1.1 catalyst balances the hydrogen adsorption and charge transfer kinetics. The above results reveal the appropriate coverage of isolated Cr_1_(OH)_x_ on Ru–Cr_1_(OH)_x_−1.1 rather than multimer chromium-oxygen clusters on heterostructure catalyst is beneficial to achieve the optimized active sites for efficient hydrogen spillover and charge transfer.

### Theoretical simulations

**Fig. 7:**
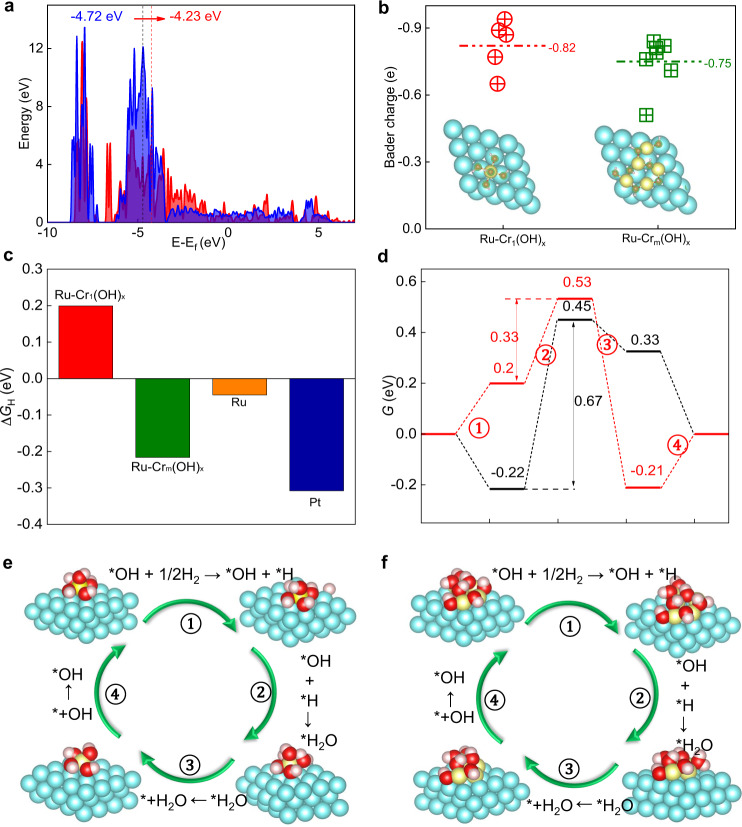
DFT calculation results. **a** DOS of O 2*p* for Ru–Cr_1_(OH)_x_ (red) and Ru–Cr_m_(OH)_x_ (blue). The dashed vertical lines represent the position of O 2*p*-band center. **b** Bader charge analysis of O atoms for Ru–Cr_1_(OH)_x_ and Ru–Cr_m_(OH)_x_. The labeled number is the average Bader charge of O atoms. **c** Free energy for H adsorption on different models. Free energy diagrams (**d**) of the elementary processes of HOR on Ru–Cr_1_(OH)_x_ (red) and Ru–Cr_m_(OH)_x_ (black) including atomic configurations of each states on Ru–Cr_1_(OH)_x_ (**e**) and Ru–Cr_m_(OH)_x_ (**f**). Color: blue, Ru; yellow, Cr; red, O; white, H.

To further verify the proposed mechanism, density functional theory (DFT) calculations were performed on the two models of Ru (001) surfaces anchored by isolated Cr_1_(OH)_x_ and multimer chromium-oxygen (Cr_m_(OH)_x_) clusters. The model surface is commonly observed and adopted both in experimental (Fig. 2b) and theoretical studies^45,46^, although it is a challenge to fully address all the surface sites under real experimental situation. The Cr_1_(OH)_x_ model is established according to the EXAFS fitting results (Supplementary Table 2) and the Cr_m_(OH)_x_ model is extracted from chromium hydroxide (Supplementary Fig. 30). The density of states (DOS) of O 2*p* indicate that the O 2*p*-band center of Ru–Cr_1_(OH)_x_ is closer to the Femi level compared with that of Ru–Cr_m_(OH)_x_, suggesting that the oxygen species of Ru–Cr_1_(OH)_x_ are more feasible to interact with the H_ad_ intermediates (Fig. 7a). The result also implies that the construction of isolated Cr_1_(OH)_x_ clusters is important to create more active oxygen species than the typical (hydr-)oxide nanoparticles, in accordance with above operando EIS investigations. The more negative charge of the oxygen species in Ru–Cr_1_(OH)_x_ further reflects the stronger basicity for capturing protons over those in Cr_m_(OH)_x_ (Fig. 7b, Supplementary Fig. 31), consistent with the NEXAFS, XPS and in situ Raman results (Figs. 4 and 6). The unique active oxygen species of Ru–Cr_1_(OH)_x_ are thus expected to interact with the adsorbed hydrogen on Ru surface and thus lower the HBE. As shown in Fig. 7c and Supplementary Fig. 32, Cr_1_(OH)_x_ can largely increase the hydrogen adsorption energy on Ru surface to 0.2 eV, which is much higher than those of Ru–Cr_m_(OH)_x_ (−0.22 eV), Ru (−0.04 eV) and Pt (−0.31 eV)^12^. With such reduced HBE induced by Cr_1_(OH)_x_, hydrogen spillover from Ru surface to Cr_1_(OH)_x_ can be easily achieved. As a result, the hydroxyl from Ru–Cr_1_(OH)_x_ reacts with the adsorbed hydrogen in the rate-determining Volmer step for *H + *OH → H_2_O with an energy barrier of only 0.33 eV (Fig. 7d-f, Supplementary Fig. 33), which is greatly decreased compared to Ru–Cr_m_(OH)_x_ (0.67 eV). The energy barrier for the Volmer step of Ru–Cr_1_(OH)_x_ is also much lower than the reported Ru or Pt-based catalysts, which usually have energy barriers higher than 1.0 eV^17,47^. All the above experimental and calculation results demonstrate that the isolated Cr_1_(OH)_x_ clusters on the Ru surface introduce unique active oxygen species, which could shuffle the H_ad_ intermediates for appropriate HBE and greatly promote hydrogen spillover from Ru to the hydroxyl of Cr_1_(OH)_x_, eventually participating in and accelerating the Volmer step of H_2_O formation for faster alkaline HOR kinetics.

In summary, constructing atomically dispersed Cr_1_(OH)_x_ on Ru surface was proposed to maximize the number of interfacial reactive sites and create a unique HOR pathway, eventually enabling efficient hydrogen oxidation. The active oxygen species activated by Cr_1_(OH)_x_ are specific for optimizing the hydrogen adsorption on Ru surface and greatly promoting hydrogen spillover from Ru to Cr_1_(OH)_x_. For further accelerated oxidation process, the original oxygen species from Cr_1_(OH)_x_ and activated oxygen species from electrolyte are both confirmed to participate in the H_2_O production of Volmer step. Meanwhile, excellent durability and CO anti-poisoning ability are also achieved because the isolated Cr_1_(OH)_x_ clusters are conducive to removing the CO molecules and supressing the aggregation of Ru species during HOR. The findings suggest that, as compared with typical metal (hydr)oxide nano-interface, atomic/cluster-level interface with unique physicochemical properties would ensure intrinsically enhanced electrocatalytic performance, which also opens a new avenue for the development of advanced catalysts via interface and/or surface engineering.

## Methods

### Synthesis of Ru–Cr_1_(OH)_x_

Ru–Cr_1_(OH)_x_ samples were prepared via a facile one-step process using NaBH_4_-induced spontaneous gelation process. Take the synthesis of Ru–Cr_1_(OH)_x_−1.1 as an example. First, 100 mg of RuCl_3_·*x*H_2_O with a 12 mg of CrCl_3_·6H_2_O were dissolved in 20 mL deionized water by an ultrasound treatment. Then NaBH_4_ aqueous solution (1 M) of 4 mL was injected into the above solution under vigorous stirring. Until no bubbles formed, the obtained precipitates were subsequently washed with water to completely remove the undeposited Cr-based precursors. Finally, the obtained solid was immediately frozen in liquid nitrogen for 10 min, and then dried under freeze drying. Ru–Cr_1_(OH)_x_−0.6 and Ru–Cr_1_(OH)_x_−2.2 were prepared by a similar procedure but using CrCl_3_·6H_2_O of 3 mg and 24 mg, respectively.

### Synthesis of Ru nanoparticle

Ru nanoparticles were synthesized following a similar procedure without addition of additional CrCl_3_·6H_2_O.

### Synthesis of ^18^O labeled Ru–Cr_1_(^18^OH)_x_ nanoparticles

First, 10 mg of RuCl_3•_xH_2_O with a 1.2 mg of CrCl_3_·6H_2_O were dissolved in 2 mL heavy-oxygen water by an ultrasound treatment. Then NaBH_4_ (0.4 mmol) was added into the above solution under vigorous stirring. Until no bubbles formed, the obtained precipitates were subsequently washed with heavy-oxygen water to completely remove the undeposited Cr-based precursors. Then, the obtained solid was immediately frozen in liquid nitrogen for 10 min, and then dried under freeze drying. Finally, the dried sample was stored under vacuum oven.

### Characterizations

The XRD patterns were collected using a RigakuDmax-rc X-ray diffractometer equipped with Cu Kα radiation (λ = 1.5418 Å). The morphologies of the samples were characterized by SEM (HITACHI SU-3500) and TEM (FEI Tecnai G^2^ F20 S-TWIN). The HAADF-STEM was performed on a FEI Titan G^2^ 60-300 equipped with a spherical aberration corrector. XPS spectra were acquired by VG Scientific ESCALab220i-XL spectrometer using Al Kα photon source equipped with 500 μm X-ray spot, and the base pressure in the analysis chamber was controlled to be 3 × 10^−10^ mbar. The Raman spectra were measured using a laser confocal fiber Raman spectrometer (Renishaw inVia-Reflex). Elemental analysis of the samples was performed with ICP-OES (ICAP-RQ, ThermoFisher Scientific, Germany). The NEXAFS spectra of the samples were collected at beamline BL10B in the National Synchrotron Radiation Laboratory (NSRL) in Hefei, China. The measurements of X-ray absorption spectroscopy were performed at beamline BL14W1 in the Shanghai Synchrotron Radiation Facility. Data processing of XANES and EXAFS was carried out with the Athena and Artemis modules of the IFEFFIT software package. The spectra were normalized, background removed and corrected according to the first and largest peak of the first derivative of XANES. *k*^3^-weighting and a *R*_bkg_ value of 1.0 were selected for the data processing. The quantitative structural parameters were obtained using a least-squares curve parameter fitting for the EXAFS spectra by ARTEMIS module. The ab initio XANES simulations of Cr K-edge XANES spectra were performed via the MXAN package, which can quantitatively analyze the XANES spectra via comparing experimental data and theoretical calculations with adjusted geometrical parameters at the photon absorber site^48^. The calculations of X-ray absorption cross-sections were carried out using the full multiple scattering approach with the muffin-tin (MT) approximation for the potential shape^49^. The optimized Cr coordinated with five oxygen atoms on Ru (001) determined by DFT calculations (Supplementary Fig. 30a) was employed as the initial structure.

### Electrochemical measurements

Electrocatalytic measurements were performed in a standard three-electrode cell system using an electrochemical station (Chi 760E). A glassy carbon electrode (GCE) with a diameter of 5 mm, graphite rod and Hg/HgO electrode were used as the working electrode, counter electrode and reference electrode, respectively. Before the electrochemical test, the GCE was consecutively polished with Al_2_O_3_ slurry with particle size of 50 nm, then washed with ethanol and water to obtain a clean surface. The catalyst ink was prepared by dispersing 0.4 mg catalysts and 1.6 mg of VulcanXC-72 in a mixed solution including 490 µL of ethanol and 10 µL of 5% Nafion solution. The above suspensions were sonicated for 1 h to obtain a homogeneous ink. Then, the electrocatalyst ink was dropped onto the GCE surface with loading of 0.06 mg_metal_ cm^−2^. After being dried, the working electrode was firstly cycled between −0.1 and −0.3 V vs. RHE at a scan rate of 200 mV s^−1^ for 5 cycles. HOR tests were carried out in H_2_-saturated KOH solution (0.1 M) with 95% iR correction and recorded at a rotation speed of 1,600 rpm with a scanning rete of 2 mV/s. The durability tests were performed in 0.1 M KOH solution by chronoamperometry experiments with carbon fiber paper (catalyst loading: ~2 mg cm^−2^) as the working electrode.

The kinetic current (*j*_K_) was calculated based on the Koutecky–Levich equation:

1 $$1/j=1/{j}_{{{{{\rm{k}}}}}}+1/{j}_{{{{{\rm{D}}}}}}$$

where *j* stands for the measured current and *j*_D_ represents the diffusion current.

The specific exchange current density (*j*_0_) was obtained by fitting *j*_K_ with Butler–Volmer equation:

2 $${j}_{{{{{\rm{k}}}}}}={j}_{0}\left[e\left(\alpha F/{{RT}}\right)\eta {{\mbox{-}}}e\left[-\left(1-\alpha \right)F/{{RT}}\right]\eta \right]$$

where *α* is the charge transfer coefficient, *R* is the universal gas constant (8.314 J mol^−1^ K^−1^), and *T* is the temperature (K), *α* and *j*_0_ were set as variables.

The electrochemical impedance spectroscopy was measured at different overpotentials and an amplitude of the sinusoidal voltage of 5 mV with a frequency range of 100 kHz to 100 mHz.

CO stripping was fitstly performed with amperometric i-t test at 0.05 V vs. RHE for 10 min in a CO-saturated 0.1 M KOH solution for CO adsorption. Then the electrode was transferred into a N_2_-saturated 0.1 M KOH solution. The The CO stripping current was obtained by CV in the potential range of 0–1.0 V vs. RHE and at a scan rate of 20 mV s^−1^.

HOR tests of Ru–Cr_1_(^18^OH)_x_ catalyst were performed in a micro electrolytic cell with the volume of 4 mL to concentrate the H_2_^18^O produced from Ru–Cr_1_(^18^OH)_x_.

### Computational details for calculations

All the spin-polarized DFT calculations on this system were performed by Vienna ab initio simulation package^50^. The projector-augmented wave (PAW) method and Perdew–Burke–Ernzerhof (PBE)^51,52^ functional within the generalized gradient approximation (GGA) were used to describe ionic cores and exchange-correlation effects, respectively. A cutoff energy of 500 eV and the Grimme method for DFT-D3 were used to account for van der Waals (vdW) interactions^52^. The vacuum gap between periodic images was set to 15 Å to avoid interaction. The convergence criteria for residual force and energy were set to 0.05 eV/Å and 10^−5^ eV, respectively. The Brillouin zone was applied by a 2 × 2 × 1 Gamma-centered special k points grid for geometry optimization. The free energy for HOR was calculated based on the computational hydrogen electrode (CHE) model proposed by Nørskov et al.^53^. In this model, the free energy of proton–electron pair is equal to that of 1/2 H_2_(g). The free energy change for each fundamental step was obtained by

3 $$\triangle G=\triangle E+{E}_{{{{{{\rm{ZPE}}}}}}}{{\mbox{-}}}T\triangle S$$

where *ΔE* is the difference of electronic energy obtained from DFT simulation. *ΔE*_ZPE_ is the variation of zero-point energy (ZPE), *ΔS* is the entropy (*S*) change, *T* is the temperature (*T* = 298.15 K). For the molecular in gas phase, the ZPE and S were taken from NIST^54^ database.

## Supplementary information

Supporting Information

## Data Availability

The data supporting the findings of this study are available within the article and its Supplementary information files. All data is available from the authors upon reasonable request.
