# Supplementary material for: Atomically dispersed chromium coordinated with hydroxyl clusters enabling efficient hydrogen oxidation on ruthenium
Source: Nat Commun. 2022 Oct 6;13:5894. doi: 10.1038/s41467-022-33625-x (PMC9537559; doi:10.1038/s41467-022-33625-x)
Supplement: Supplementary file 1 — Supporting Information [file 41467_2022_33625_MOESM1_ESM.pdf]

Atomically Dispersed Chromium Coordinated with Hydroxyl Clusters Enabling Efficient Hydrogen Oxidation on Ruthenium

Zhang et al.

Supplementary Figs.

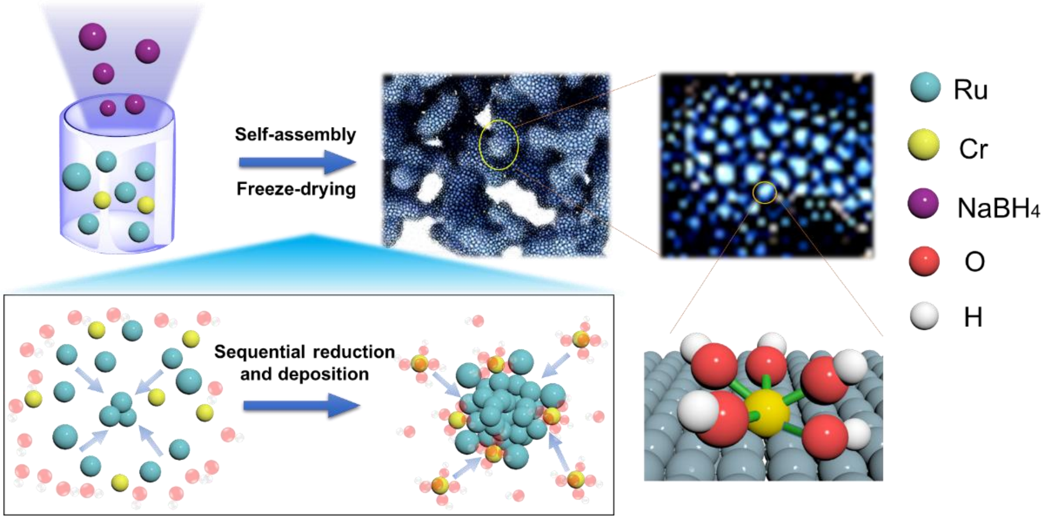

Supplementary Fig. 1 | Schematic illustration of the synthesis and structure of Ru-$Cr_1(OH)_x$.

The driving force for the formation of the $Cr_1(OH)_x$ clusters on metallic Ru surface primarily stems from the strong reduction and basic conditions during the synthesis process. Owing to the more negative standard electrode potential of Cr^{3+} relative to Ru^{3+} ($Cr^{3+} + 3e^- \rightleftharpoons Cr$, E^0 = -0.74 V vs. SHE; $Ru^{3+} + 3e^- \rightleftharpoons Ru$, E^0 = 0.79 V vs. SHE), the reduction of Ru^{3+} takes place first to form metallic Ru nanoparticles while the Cr^{3+} species cannot be reduced to metallic state under the same reduction condition. $NaBH_4$ hydrolysis would induce strong basic condition, generating high-concentration of hydroxyl, and consequently, the Cr^{3+} can be easily coordinated with the hydroxyl ligands to form the $Cr_1(OH)_x$ clusters, which are deposited on Ru surface simultaneously.

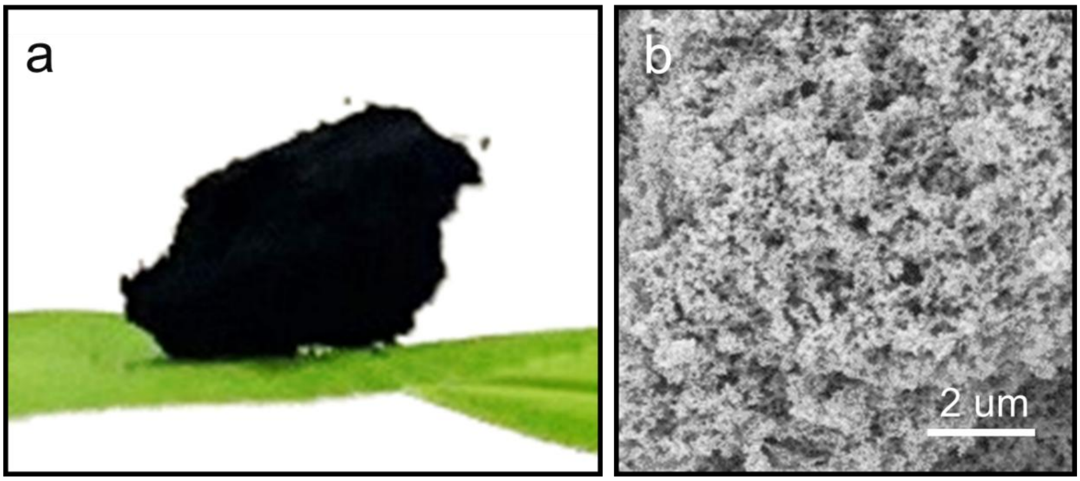

Supplementary Fig. 2 | Morphology of Ru-$Cr_1(OH)x$. Photograph (**a**) and SEM image (**b**) of Ru-$Cr_1(OH)_x$ aerogel catalyst.

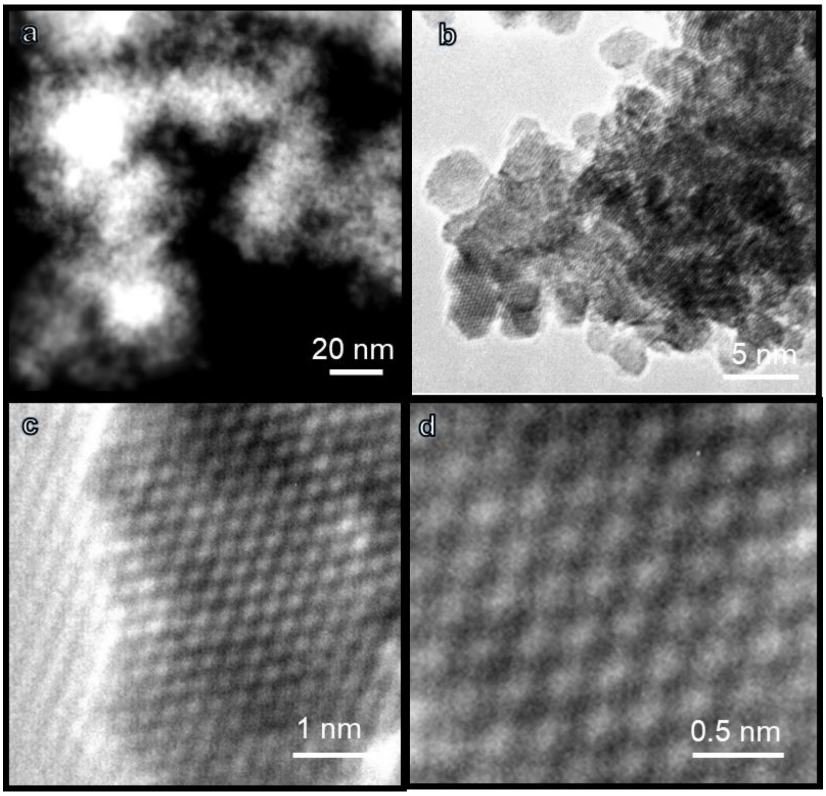

Supplementary Fig. 3 | Additional characterizations for Ru-$Cr_1(OH)_x$. a HAADF-STEM image. **b-c** HRTEM images.

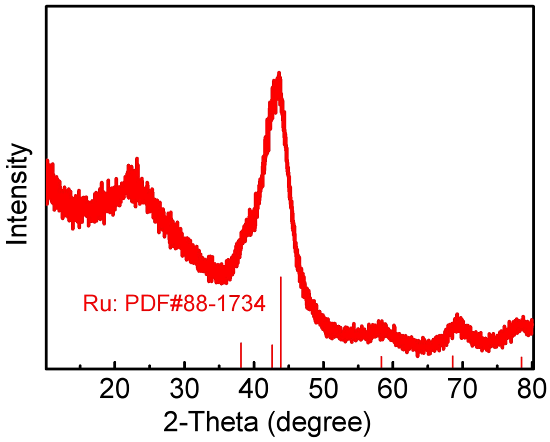

Supplementary Fig. 4 | XRD pattern of Ru-$Cr_1(OH)_x$.

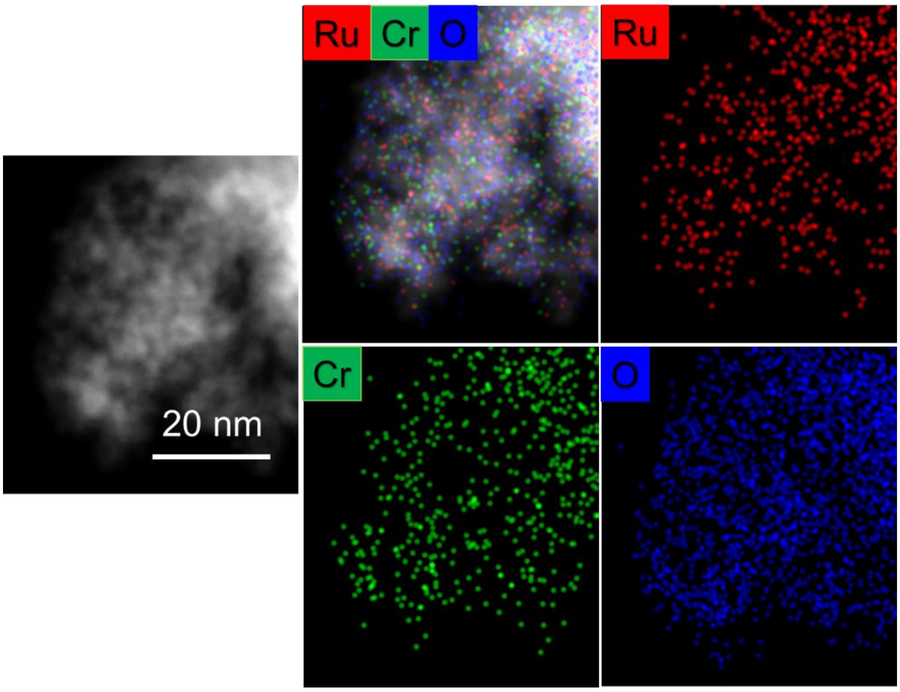

Supplementary Fig. 5 | EDS element mapping images of Ru-$Cr_1(OH)_x$.

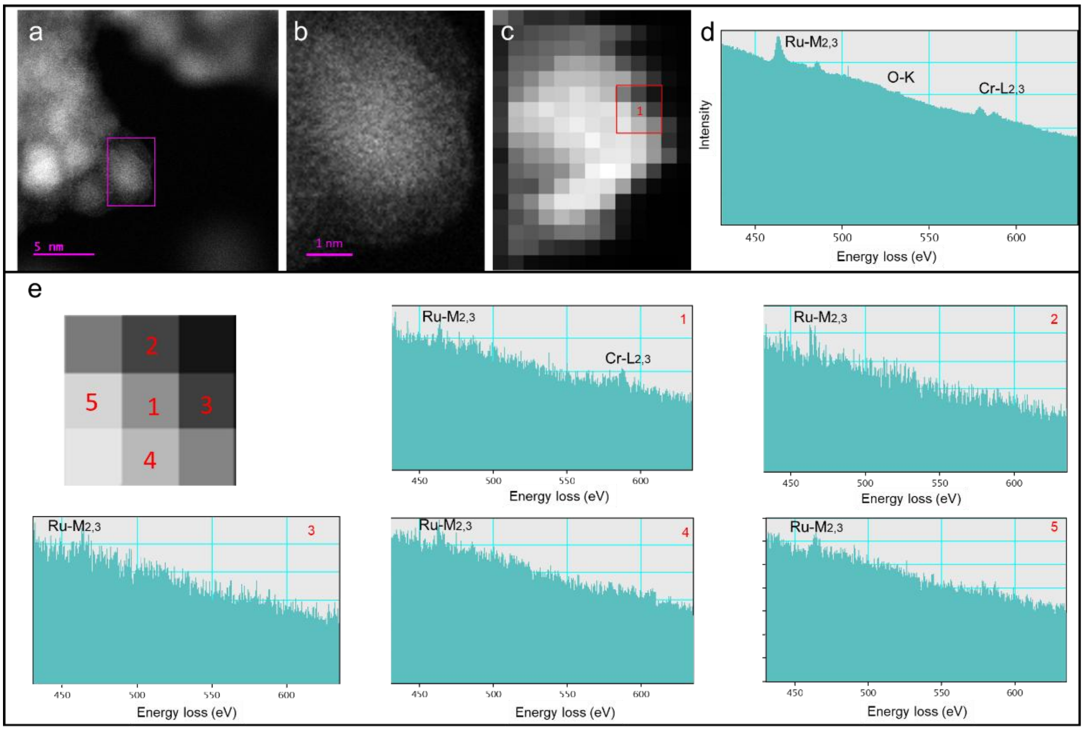

Supplementary Fig. 6 | EELS map scanning image of Ru-$Cr_1(OH)_x$. a HAADF-STEM image. **b,c** Enlarged HAADF-STEM image (**b**) and corresponding EELS map scanning image (**c**). **d** The total EELS of the map in (c). **e** The enlarged image of the area in the red square of (c) and corresponding EELS spectra of site 1 and its adjacent sites.

The local pixels in Supplementary Fig. 6c are enlarged in Supplementary Fig. 6e, where the serial numbers match with that of extracted EELS spectrums. Thereinto, the signal peak of Cr L_3 edge is only detected in the central pixel 1, implying that the Cr species are atomically dispersed at the Ru support.

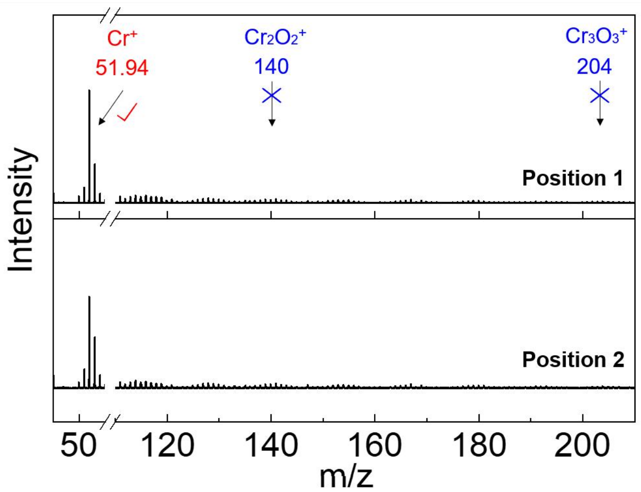

Supplementary Fig. 7 | ToF-SIMS (+) spectra of the Ru-$Cr_1(OH)_x$-1.1 sample detected on different positions.

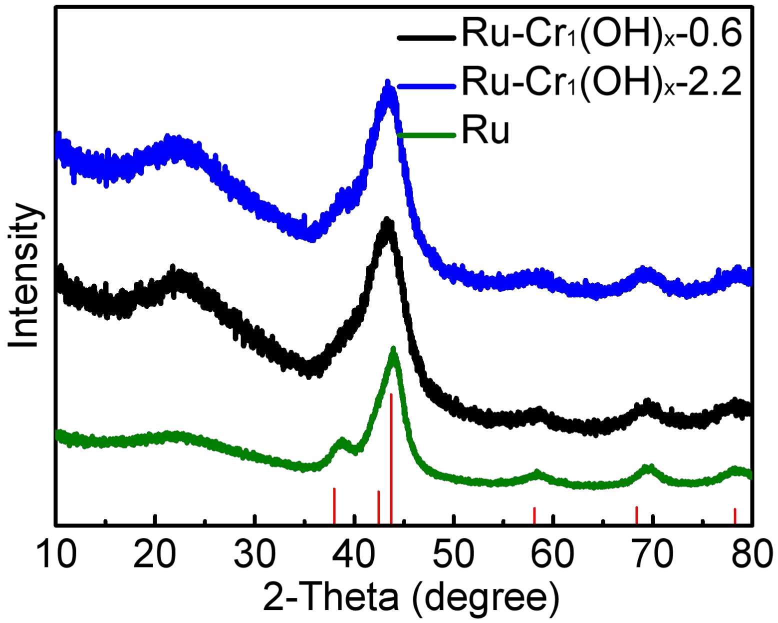

Supplementary Fig. 8 | XRD patterns of Ru nanoparticles, Ru-$Cr_1(OH)_x$-0.6 and Ru-$Cr_1(OH)_x$-2.2.

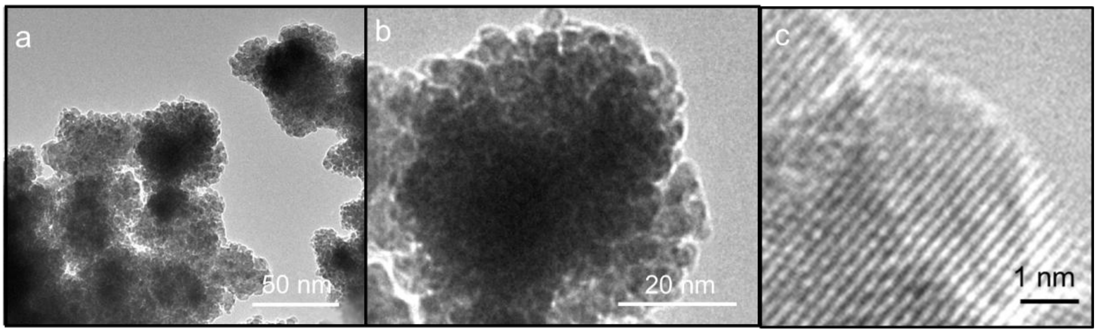

Supplementary Fig. 9 | Structural characterizations of Ru nanoparticles. TEM (**a,b**) and HRTEM (**c**) images.

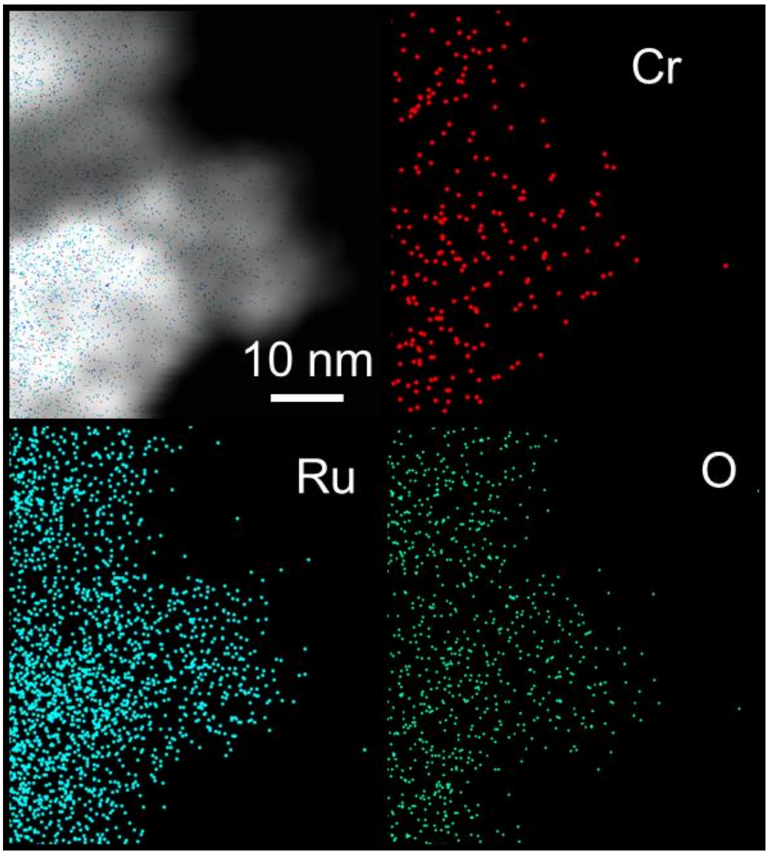

Supplementary Fig. 10 | EDS elemental mappings of Ru-$Cr_1(OH)_x$-0.6.

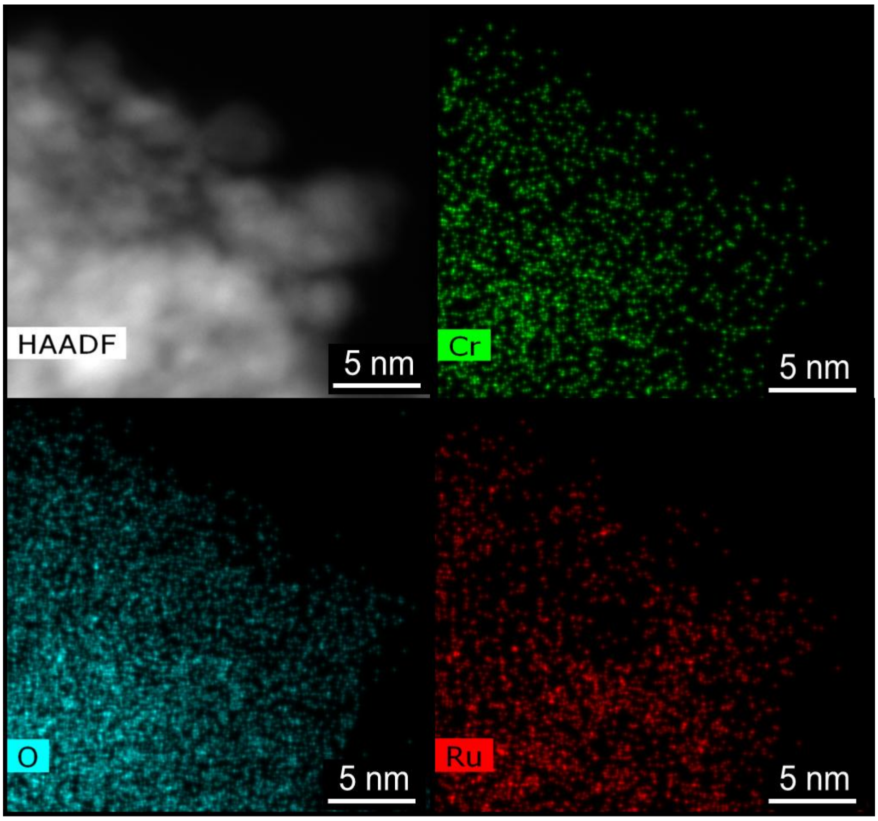

Supplementary Fig. 11 | EDS elemental mappings of Ru-$Cr_1(OH)_x$-2.2.

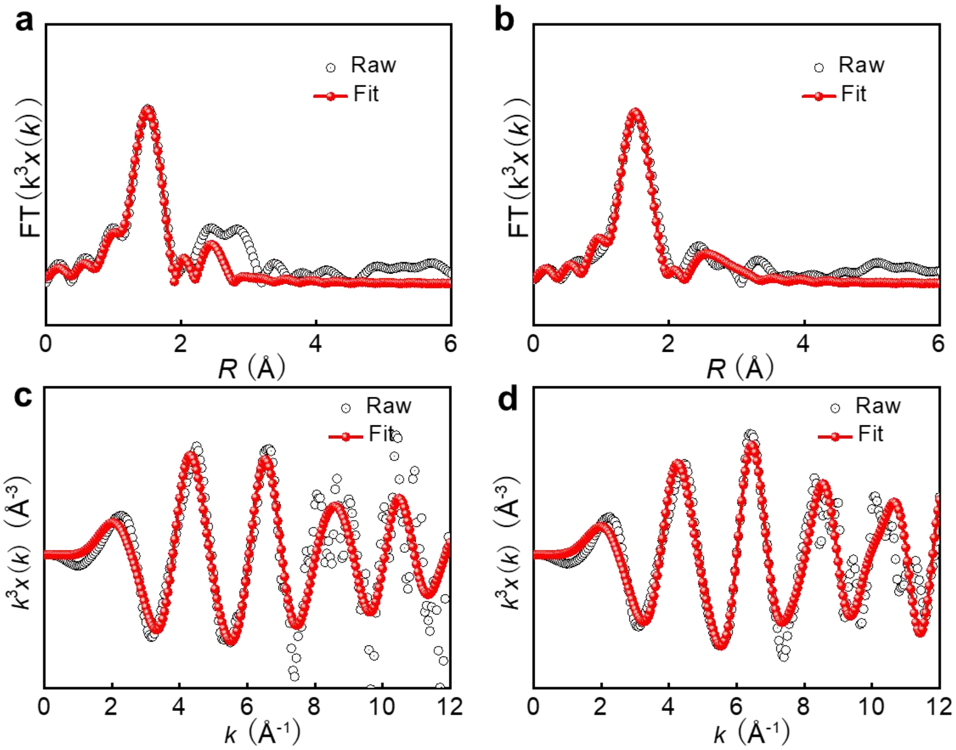

Supplementary Fig. 12 | EXAFS fitting results. EXAFS fitting curves of Ru-$Cr_1(OH)_x$-0.6 in *R* (**a**) and *k* (**c**) space and Ru-$Cr_1(OH)_x$-2.2 in *R* (**b**) and *k* (**d**) space.

The features past 2 Å are assigned to the interactions of Cr-Ru (2.42 Å) and Cr-O-Cr (2.54 Å). The features past 2.7 Å may be attributed to the interactions between Cr atom and the other Ru atoms from Ru substrate.

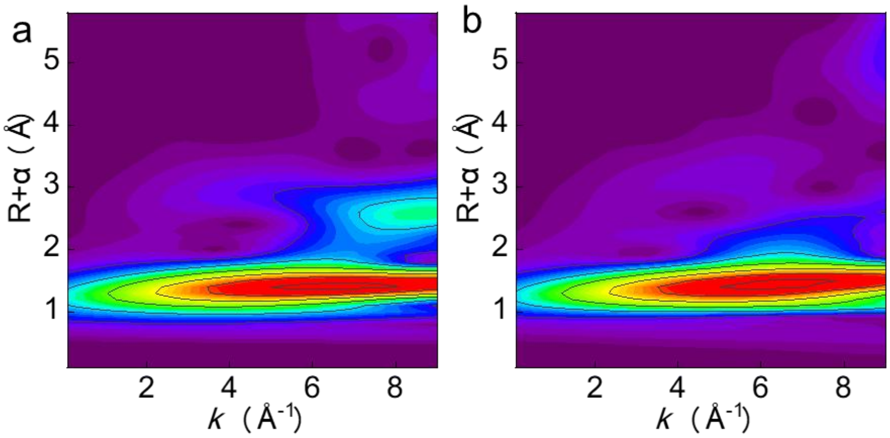

Supplementary Fig. 13 | WT plots. WT for the k^3-weighted EXAFS of Ru-$Cr_1(OH)_x$-0.6 in *R* (**a**) and Ru-$Cr_1(OH)_x$-2.2 (**b**).

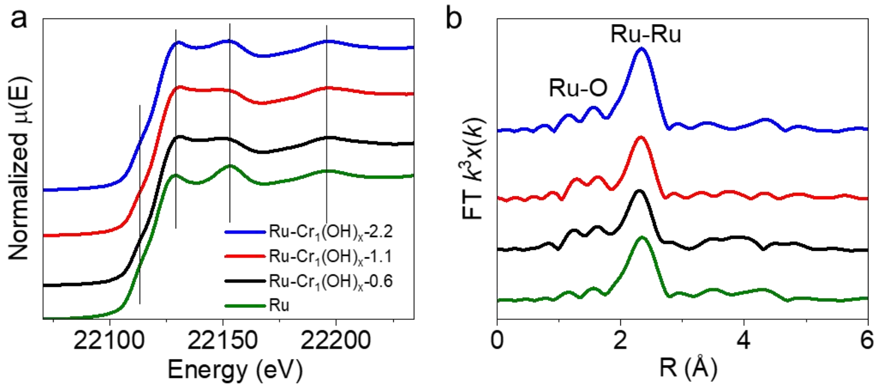

Supplementary Fig. 14 | XAFS measurements of different samples at the Ru K edge. a XANES spectra. **b** k^3-weighted FT-EXAFS spectra in *R*-space. Inset in (**b**) is the calculated and optimized model of $Cr_1(OH)_x$ loaded on Ru surface.

The relative intensity variation of the two main peaks and extra EXAFS scattering intensity below 2 Å are possibly related to the additional Ru-O scattering path originated from some oxidized Ru species and $Cr_1(OH)_x$, but this will not affect the main metallic Ru surface as reflected in the HAADF-STEM images (Fig. 2b, Supplementary Fig. 3).

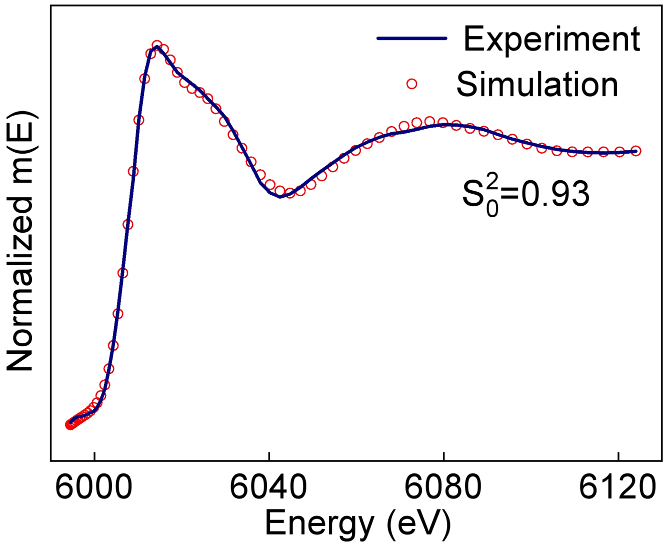

Supplementary Fig. 15 | XANES simulation. Comparison between the experimental (solid line) and the simulated (dotted line) XANES spectra for Ru-$Cr_1(OH)_x$-1.1.

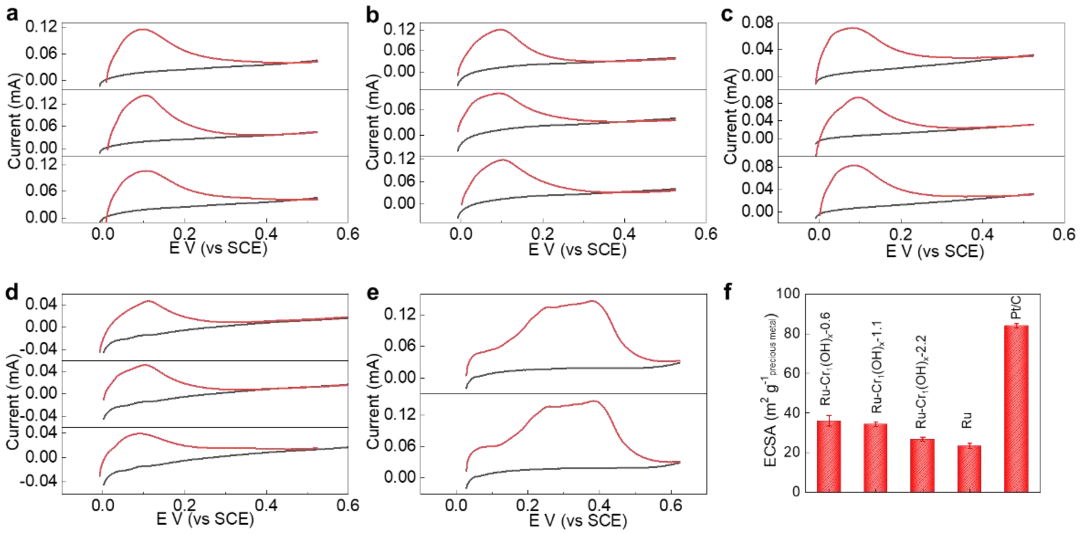

Supplementary Fig. 16 | ECSA test. Estimation of the ECSA using the copper underpotential deposition (Cu-UPD) method for Ru-$Cr_1(OH)_x$-0.6 (**a**), Ru-$Cr_1(OH)_x$-1.1 (**b**), Ru-$Cr_1(OH)_x$-2.2 (**c**), Ru NP (**d**), Pt/C (**e**) and summary of the ECSA values for different catalysts (**f**). Voltammogram curves were acquired in 0.5 M H_2SO_4 solutions in the absence (black) and presence (red) of 5 mM $CuCl_2$. Three tests were performed to evaluate the ECSA for each catalyst. The ECSA of Ru-$Cr_1(OH)_x$ catalysts is slightly decreased with the increase of Cr anchoring. The Ru-$Cr_1(OH)_x$ catalysts show higher ECSA values than that of Ru NPs because the appropriate amount of Cr anchoring can suppress the agglomeration of Ru NPs during the synthesis process (Supplementary Figs. 3 and 9).

For Cu-UPD test, to obtain monolayered Cu deposited on metal, the Pt/C and Ru-based electrodes were polarized at underpotentially deposited potentials for 100 s in an Ar-purged 0.5 M H_2SO_4 solution containing 5 mM of $CuCl_2$. Then, the Cu-UPD stripping voltammetry was performed by oxidizing the deposited Cu between the underpotentially deposited potential and 0.7 V at a scan rate of 10 mV s^{-1} (red curves). The voltammogram curve (black curve) of each catalyst in 0.5 M H_2SO_4 solution without $CuCl_2$ was applied as the background for the corresponding Cu-UPD stripping voltammogram. The ECSA was evaluated from the integral area of Cu-UPD peaks (Q_{Cu}) with the subtraction of the background and a charge density of 420 μC cm^{-2} (Q_s):

$$\mathrm{ECSA}\ (\mathrm{m}^2_{\mathrm{metal}}/\mathrm{g}_{\mathrm{metal}}) = Q_{Cu} / (M_{\mathrm{metal}} * Q_s)$$

where M_{metal} is the metal mass loading on a certain geometric area of the working electrode.

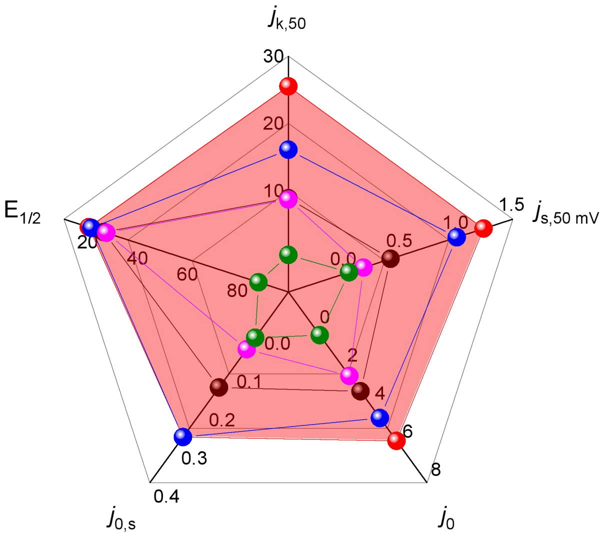

Supplementary Fig. 17 | Performance comparison of different electrocatalysts for HOR. Ru-$Cr_1(OH)_x$-1.1 (red), Ru-$Cr_1(OH)_x$-2.2 (blue), Ru-$Cr_1(OH)_x$-0.6 (black), Pt/C (pink) and Ru NP (green).

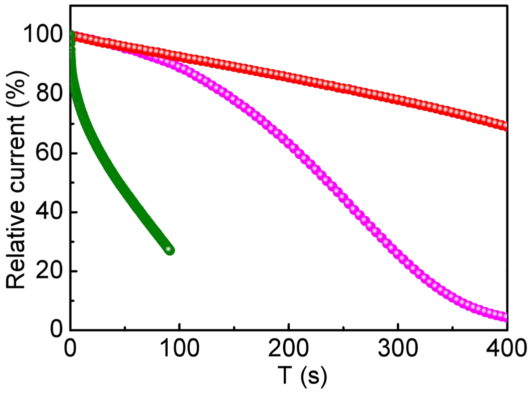

Supplementary Fig. 18 | Anti-poisoning test of CO. Relative current-time chronoamperometry response of Ru-$Cr_1(OH)_x$-1.1 (red), Ru NP (green) and Pt/C (pink) in H_2-saturated 0.1 M KOH with the presence of CO at 0.05 V versus RHE.

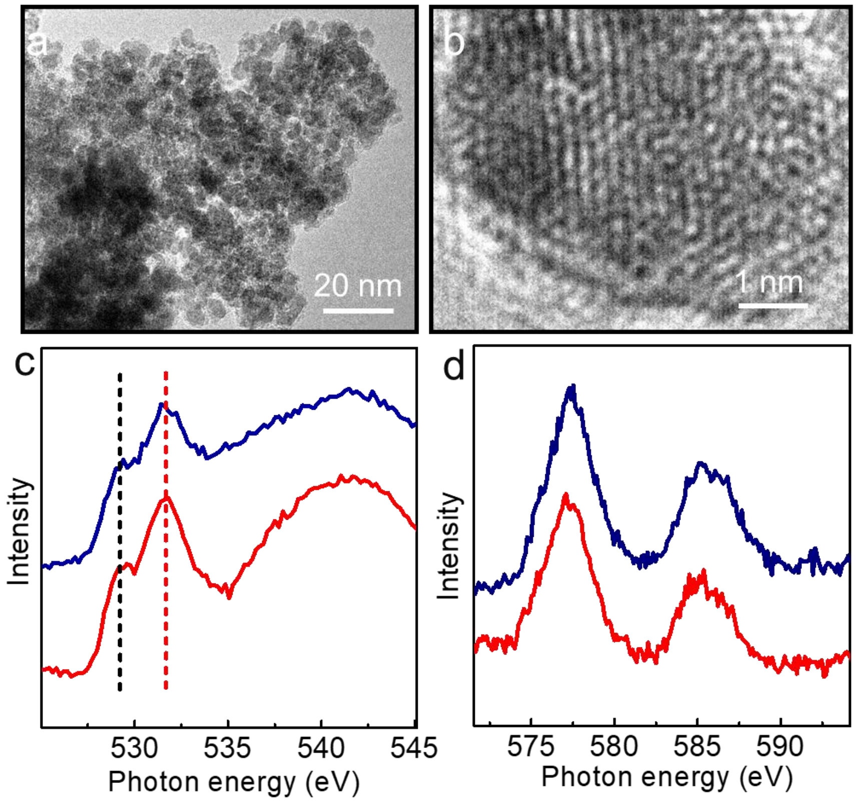

Supplementary Fig. 19 | Characterizations of Ru-$Cr_1(OH)_x$-1.1 after stability test. **a,b** TEM (**a**) and HRTEM (**b**) images. **c,d** O K (**c**) and Cr $L_{II,\ III}$ (**d**) and edges NEXAFS spectra for Ru-$Cr_1(OH)_x$-1.1 before (red) and after (dark blue) stability test.

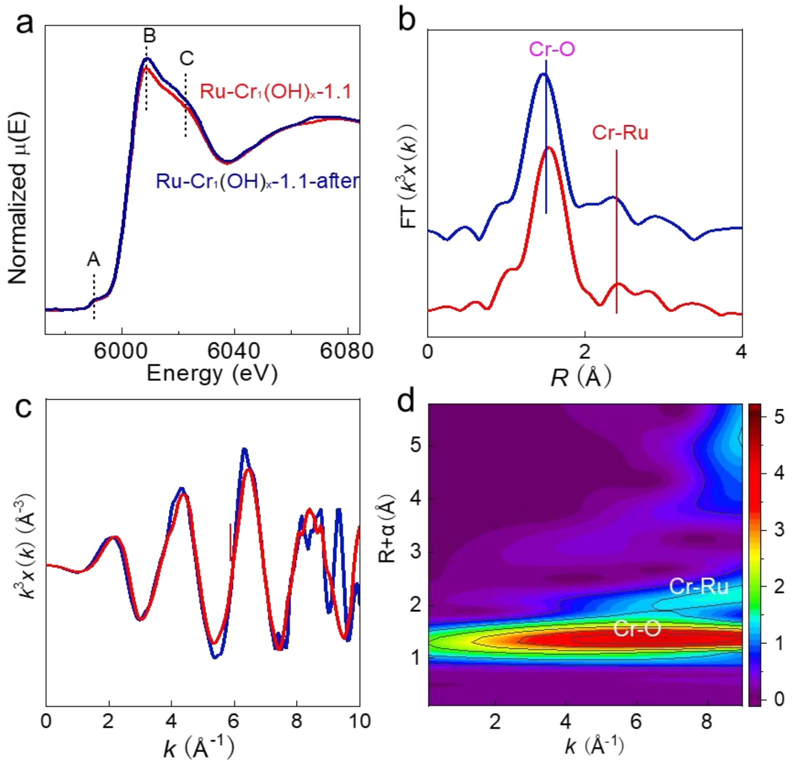

Supplementary Fig. 20 | XAFS Characterizations of Ru-$Cr_1(OH)_x$-1.1 after stability test. a XANES spectra. **b,c** k^3-weighted FT-EXAFS spectra in R-space (b) and k-space (c). **d** WT for the k^3-weighted EXAFS of Ru-$Cr_1(OH)_x$-1.1 after stability test.

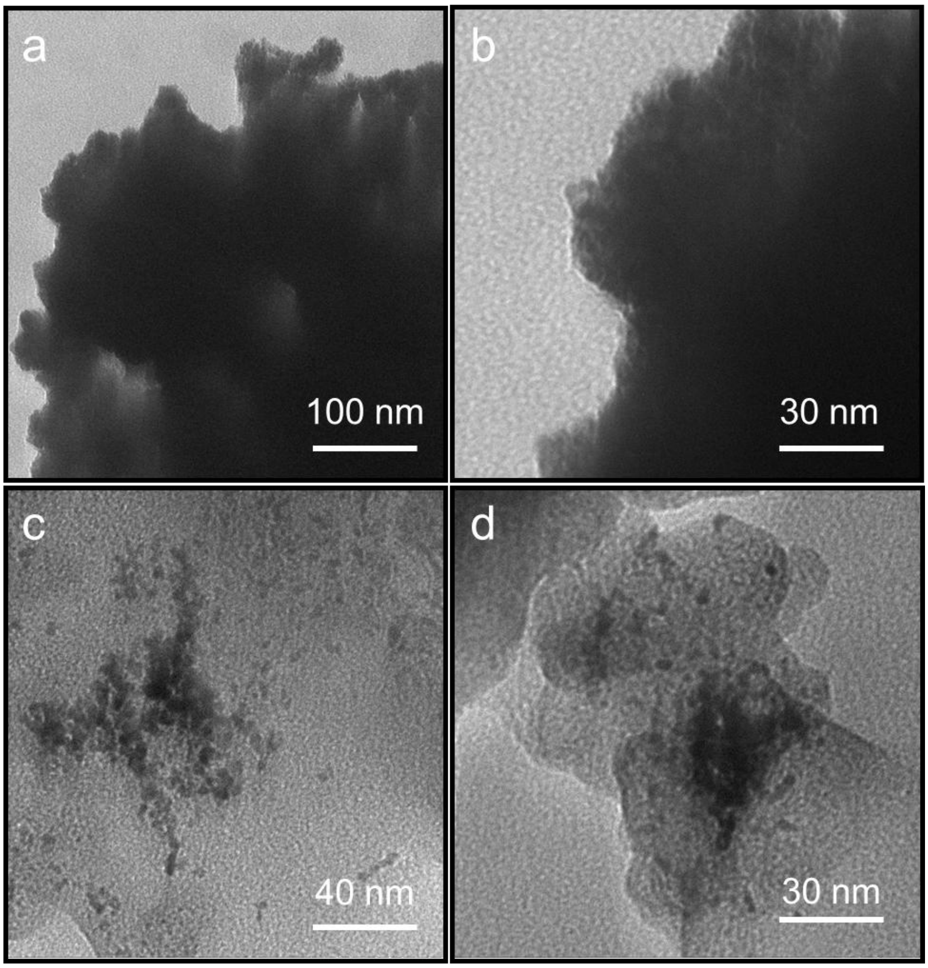

Supplementary Fig. 21 | Characterizations of Ru NPs and Pt/C after stability test.

TEM images of Ru NPs (**a,b**) and Pt/C (**c,d**).

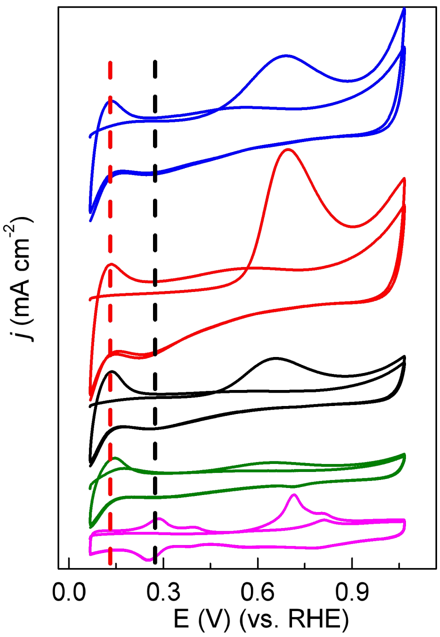

Supplementary Fig. 22 | CO-stripping voltammograms. CO-stripping voltammograms of Ru-$Cr_1(OH)_x$-0.6 (black), Ru-$Cr_1(OH)_x$-1.1 (red), Ru-$Cr_1(OH)_x$-2.2 (blue), Ru NP (green) and Pt/C (pink).

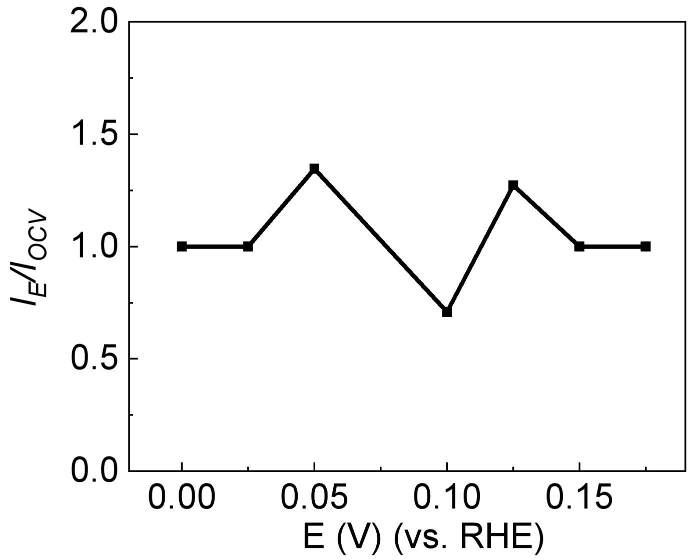

Supplementary Fig. 23 | Peak intensity ratios of in situ Raman spectra. The peak intensity ratios of Cr-O-H bending mode in situ Raman spectra of Ru-$Cr_1(OH)_x$-1.1 at different overpotentials. I_E is the intensity at different applied potential and I_{OCV} is the initial intensity at OCV. The peak intensity ratios seem to be fluctuant in the high kinetic region at higher potentials maybe due to the accelerated vibrations of reaction conditions containing local gas, ions and intermediate adsorption.

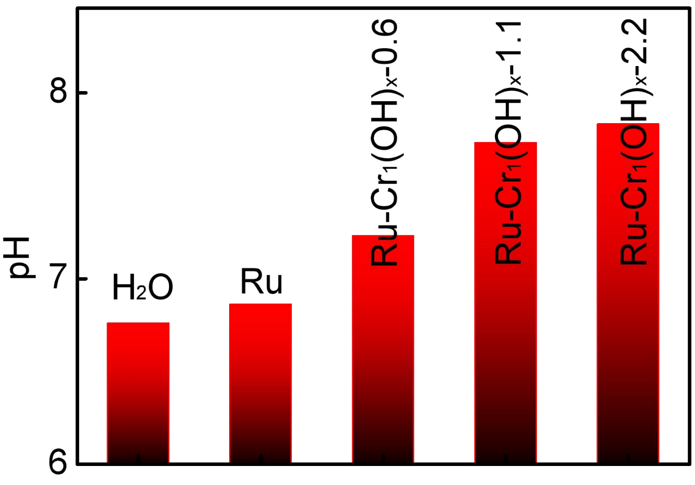

Supplementary Fig. 24 | pH changes of nanopure water after dispersing Ru-$Cr_1(OH)_x$ powders. The pH change of nanopure water before and after dispersing catalyst powders (3 mg/mL).

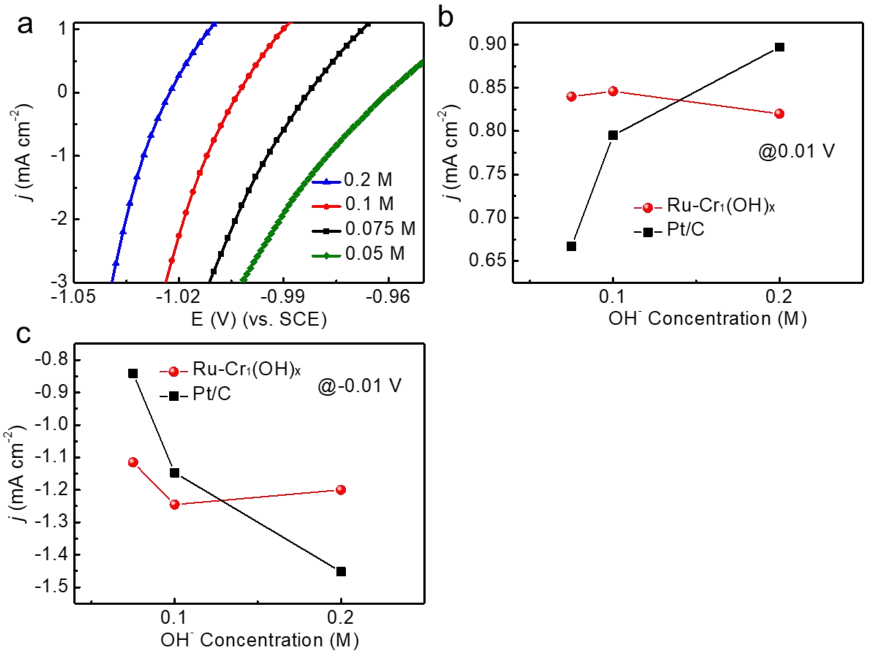

Supplementary Fig. 25 | Electrochemical performances in different pH conditions. a LSV curves of Pt/C for HOR in different pH conditions. **b,c** The current densities at 0.01 V and -0.01 V (vs. RHE) in different OH^- concentrations.

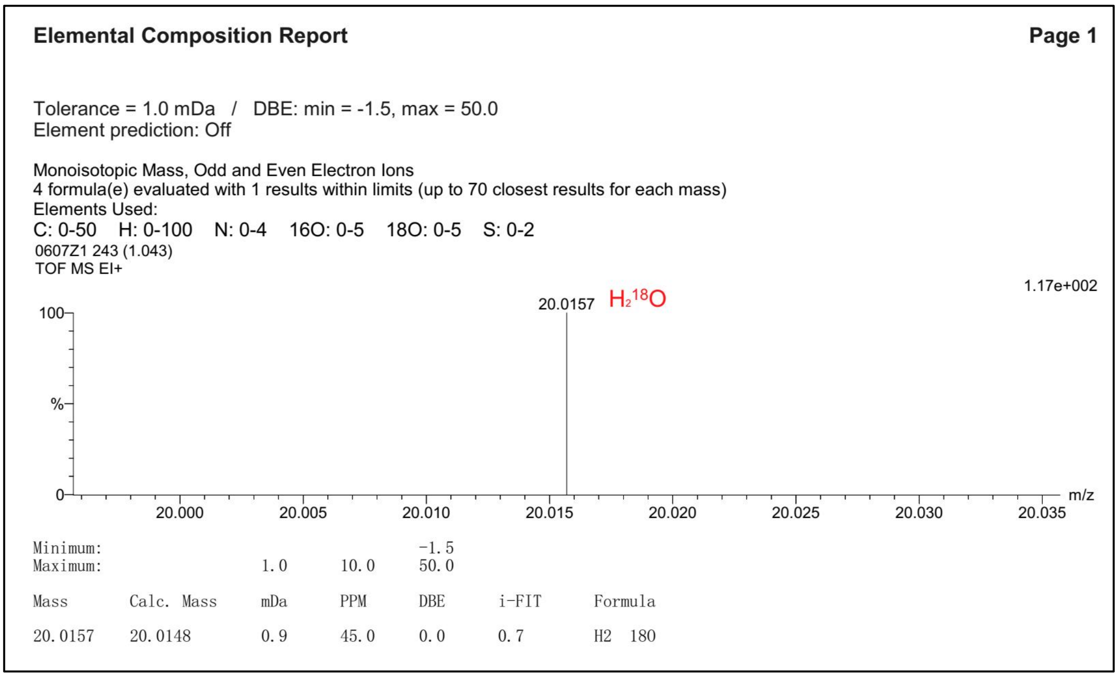

Supplementary Fig. 26 | Detection of reaction product. Time-dependent mass spectra of the electrolyte after HOR catalyzed by Ru-$Cr_1(^{18}OH)_x$.

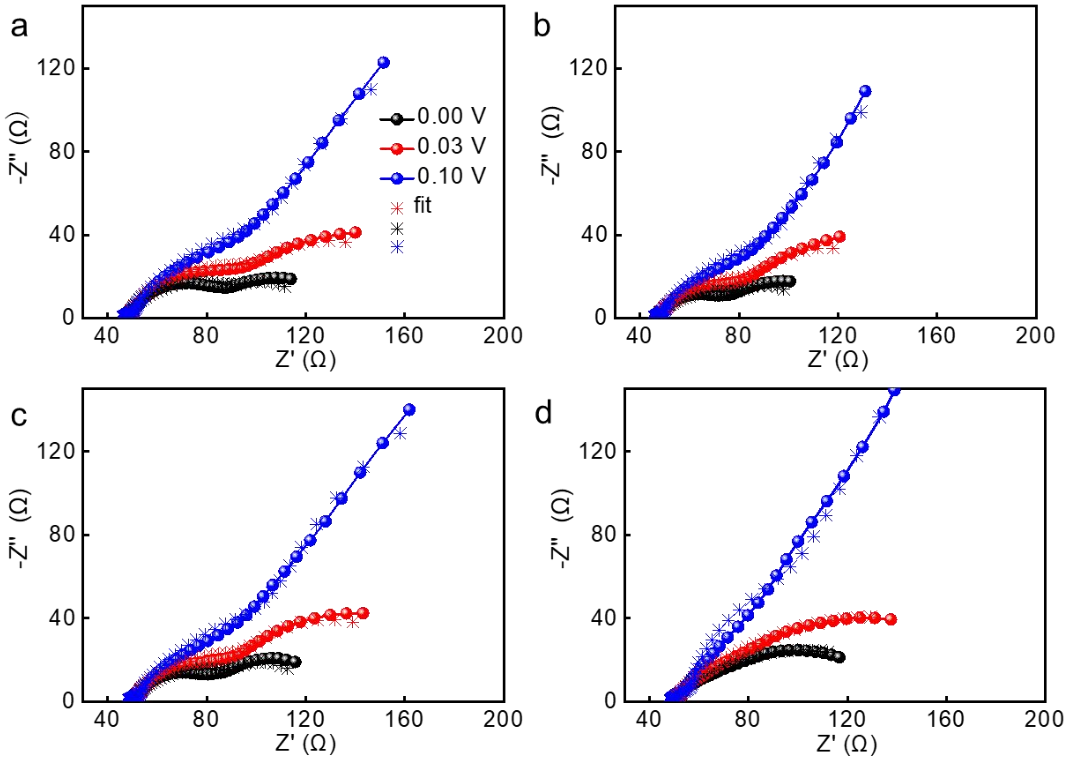

Supplementary Fig. 27 | Operando EIS. Operando EIS with simulated fitting results at different operated potentials for Ru-Cr$_1$(OH)$_x$-0.6 (a), Ru-Cr$_1$(OH)$_x$-1.1 (b), Ru-Cr$_1$(OH)$_x$-2.2 (c) and Pt/C (d).

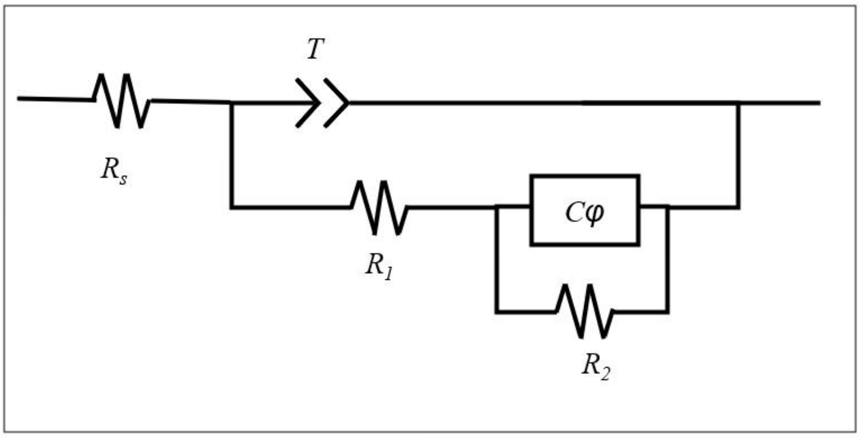

Supplementary Fig. 28 | Equivalent circuit applied to simulate the EIS plots.

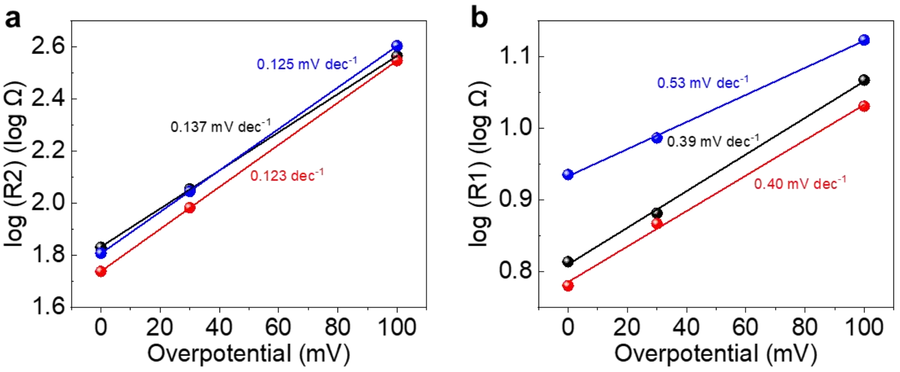

Supplementary Fig. 29 | EIS-derived Tafel plots. EIS-derived Tafel plots of the Ru-$Cr_1(OH)_x$-0.6 (black), Ru-$Cr_1(OH)_x$-1.1 (red) and Ru-$Cr_1(OH)_x$-2.2 (blue) catalysts obtained from the hydrogen adsorption resistance R_2 (**a**) and charge transfer resistance R_1 (**b**).

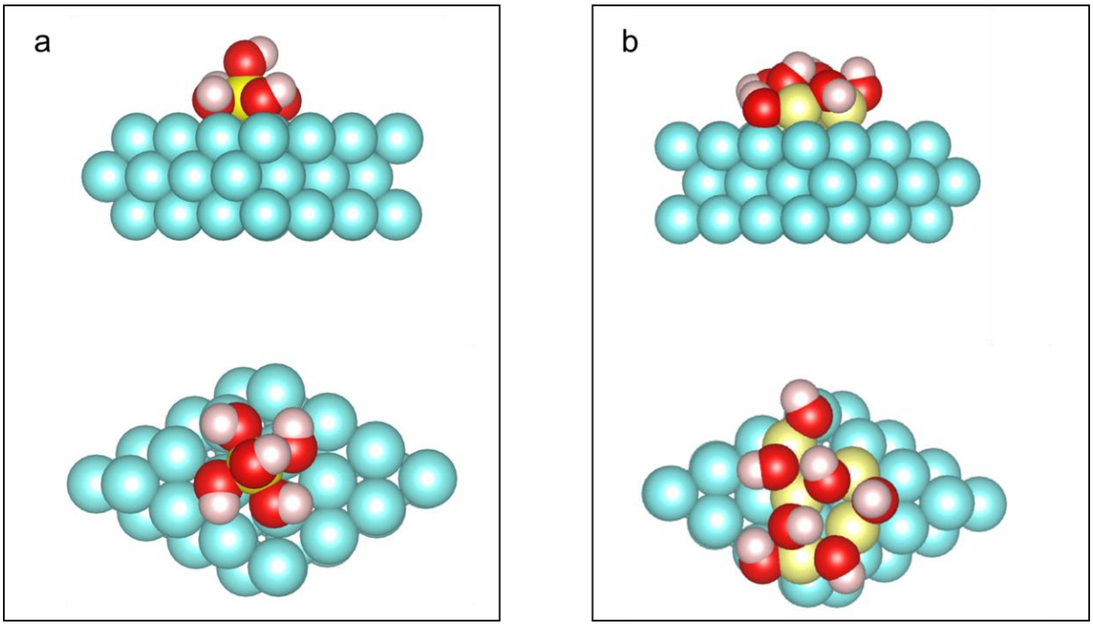

Supplementary Fig. 30 | Models of Ru-$Cr_1(OH)_x$ and Ru-$Cr_m(OH)_x$. a,b Optimized atomic structure of Ru-$Cr_1(OH)_x$ (**a**) and Ru-$Cr_m(OH)_x$ (**b**) in side view (top) and top view (bottom). Color: blue, Ru; yellow, Cr; red, O; white, H.

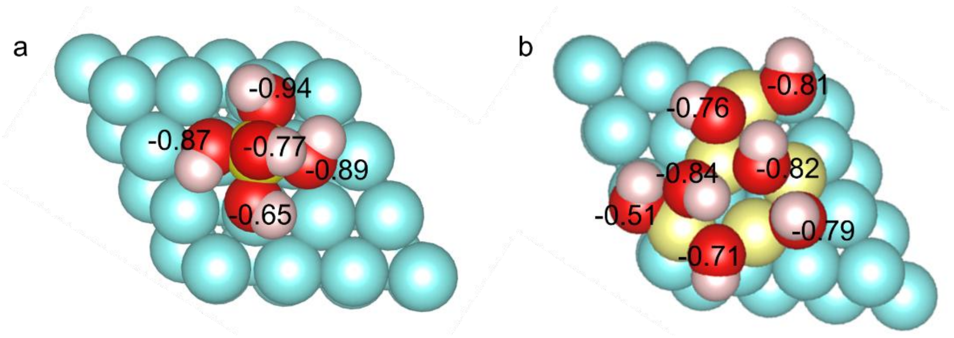

Supplementary Fig. 31 | Bader charge analysis. Bader charge analysis of O atoms for Ru-$Cr_1(OH)_x$ (a) and Ru-$Cr_m(OH)_x$ (b).

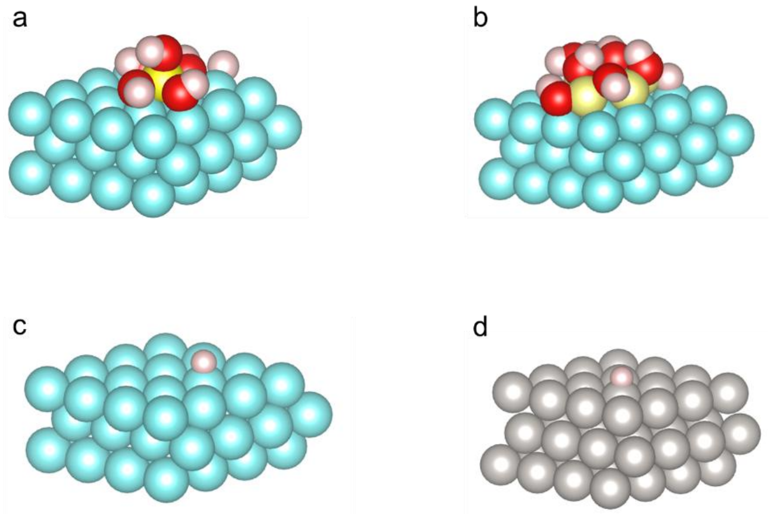

Supplementary Fig. 32 | Models for hydrogen adsorption. Optimized atomic structure for hydrogen adsorption of Ru-$Cr_1(OH)_x$ (**a**), Ru-$Cr_m(OH)_x$ (**b**), Ru (**c**) and Pt (**d**). Color: blue, Ru; yellow, Cr; red, O; white, H; grey, Pt.

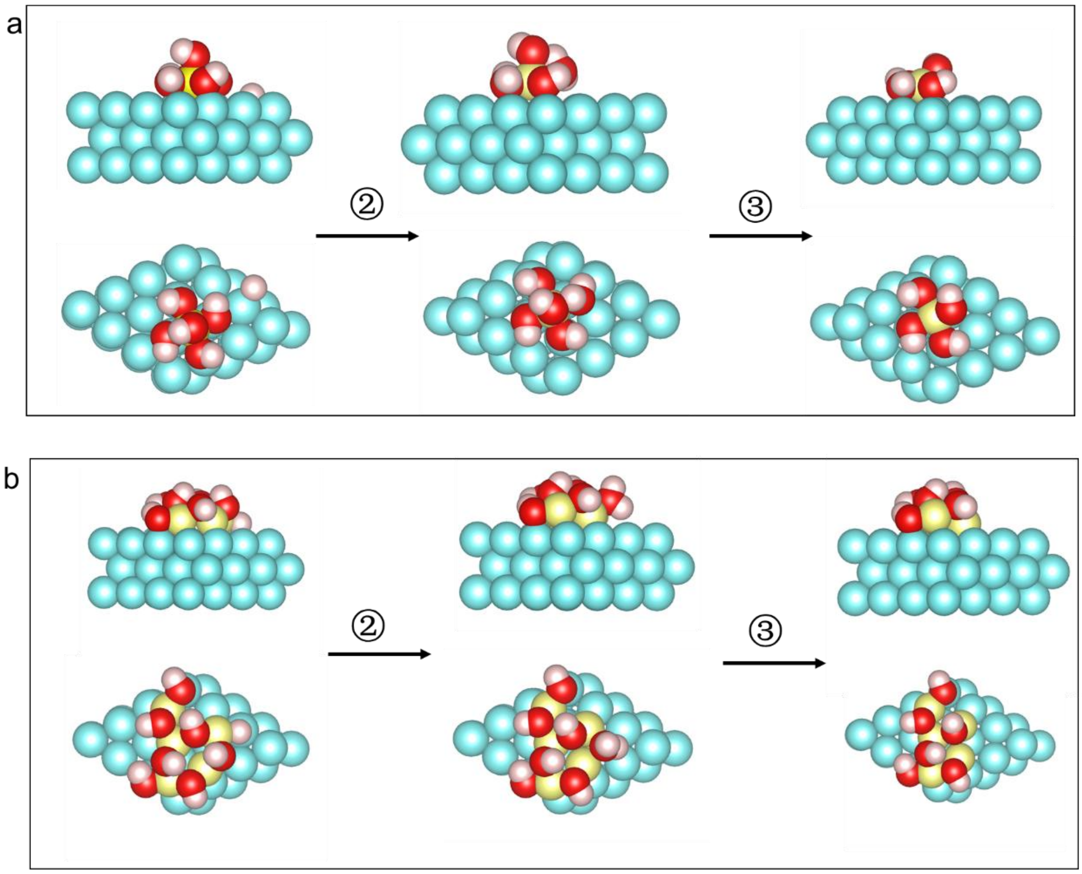

Supplementary Fig. 33 | Atomic configurations for different reaction states. Side view and top view of the detail atomic configurations on Ru-$Cr_1(OH)_x$ (**a**) and Ru-$Cr_m(OH)_x$ (**b**) corresponding to Fig. 6d in the main text. Color: blue, Ru; yellow, Cr; red, O; white, H.

Supplementary Table 1. Chemical compositions of Ru-$Cr_1(OH)_x$ catalysts determined by XPS and ICP.

Sample	Cr (wt%)[a]	Cr (at%)[b]
Ru-$Cr_1(OH)_x$-0.6	0.6	2.3
Ru-$Cr_1(OH)_x$-1.1	1.1	4.9
Ru-$Cr_1(OH)_x$-2.2	2.2	10.7

[a]The Cr content in the catalysts obtained from ICP; [b]The relative content of Cr (Cr/Cr+Ru) on the catalyst surface obtained from the XPS.

Supplementary Table 2 | Best fitting EXAFS data for different Cr samples.

Sample	Path	CN	R/Å	σ^2/x10^{-3} Å	ΔE_0/eV	X_{Cr1}
Cr foil	Cr-Cr	12	2.56			
Cr_2O_3	Cr-O	3	2.01			
	Cr-O	3	2.06			
	Cr-Cr	1	2.72			
	Cr-Cr	3	2.96			
$Cr(OH)_3$	Cr-O	3	2.01			
	Cr-O	3	2.05			
	Cr-Cr	6	3.06			
Ru-$Cr_1(OH)_x$-0.6	Cr-O	5.0	1.96	0.0045	-2.6	93%
	Cr-Ru	1.7	2.72	0.0120	-2.1	
	Cr-Cr	0.4	3.11	0.0088	-2.1	
Ru-$Cr_1(OH)_x$-1.1	Cr-O	4.5	1.97	0.0017	-3.9	90%
	Cr-Ru	1.5	2.67	0.0150	-2.5	
	Cr-Cr	0.6	3.04	0.0021	-2.5	
Ru-$Cr_1(OH)_x$-2.2	Cr-O	4.7	1.96	0.0033	-3.1	82%
	Cr-Ru	1.5	2.68	0.009	-2.8	
	Cr-Cr	1.1	3.05	0.002	-2.8	

CN is the coordination number; *R* is interatomic distance (the bond length between central atoms and surrounding coordination atoms); σ^2 is Debye-Waller factor (a measure of thermal and static disorder in absorber-scatterer distances); ΔE_0 is edge-energy shift (the difference between the zero kinetic energy value of the sample and that of the theoretical model). Error bounds that characterize the structural

parameters obtained by EXAFS spectroscopy were estimated as N ± 20%; R ± 1%; σ^2 ± 20%; ΔE_0 ± 20%. X_{Cr1} is the fraction of isolated Cr species in the sample, which can be calculated based on the equation:

$X_{Cr1} = [1 - (N_{Cr\text{-}Cr}/N_{chromium\text{-}hydroxide\text{-}cluster})]\times 100\%$

$N_{Chromium\text{-}hydroxide\text{-}cluster}$ is the Cr-Cr CNs in chromium hydroxide clusters, in which the multimer chromium-oxygen clusters are 6 in the bulk structure. The fraction of isolated Cr_1 species in Ru-$Cr_1(OH)_x$-1.1 could be determined to be $XCr_1 \approx [1-(0.6/6)]\times 100\% = 90\%$.

Supplementary Table 3 | Performance comparison of various electrocatalysts for HOR.

**Catalyst**	**ECSA (m^2 g^{-1})**	**$j_{k,50\ mV}$ (mA cm^{-2})**	**$j_{s,50\ mV}$ (A cm_{PGM}^{-2})**	**j_0 (mA cm^{-2})**	**$j_{0,s}$ (mA cm_{PGM}^{-2})**	**$E_{1/2}$ (V vs. RHE)**
**Ru-$Cr_1(OH)_x$-0.6**	36.1	8.9	0.41	3.2	0.15	26
**Ru-$Cr_1(OH)_x$-1.1**	34.2	25.5	1.24	5.8	0.28	20
**Ru-$Cr_1(OH)_x$-2.2**	26.8	16.1	1.00	4.6	0.28	21
**Pt/C**	82.8	8.7	0.17	2.4	0.05	27
**Ru NP**	23.6	0.5	0.04	0.26	0.02	88

Supplementary Table 4 | EIS fitting results.

Catalyst	η (mV)	R_s (Ω)	T (F s^{n-1})	R_1 (Ω)	R_2 (Ω)	$C_φ$ (F)
Ru-$Cr_1(OH)_x$-0.6	0	46.09	0.004172	6.51	67.79	0.000252
	30	46.13	0.00469	7.61	113.40	0.000297
	100	46.16	0.00574	11.68	367.70	0.00041
Ru-$Cr_1(OH)_x$-1.1	0	45.77	0.000618	6.03	54.71	0.000165
	30	45.76	0.006674	7.36	96.11	0.000196
	100	45.77	0.006955	10.74	353.10	0.000369
Ru-$Cr_1(OH)_x$-2.2	0	48.45	0.004858	8.62	64.24	0.00014
	30	48.43	0.005197	9.70	111.00	0.000176
	100	48.3	0.005478	13.29	402.40	0.000318
Pt/C	0	48.32	0.00813	14.35	101.60	0.000335
	30	48.23	0.007763	15.37	185.70	0.000347
	100	48.23	0.008187	21.80	577.50	0.005333
